# *Plasmodium* 6-Cysteine Proteins: Functional Diversity, Transmission-Blocking Antibodies and Structural Scaffolds

**DOI:** 10.3389/fcimb.2022.945924

**Published:** 2022-07-08

**Authors:** Frankie M. T. Lyons, Mikha Gabriela, Wai-Hong Tham, Melanie H. Dietrich

**Affiliations:** ^1^ The Walter and Eliza Hall Institute of Medical Research, Infectious Diseases and Immune Defence Division, Parkville, VIC, Australia; ^2^ Department of Medical Biology, The University of Melbourne, Melbourne, VIC, Australia

**Keywords:** monoclonal antibodies, 6-cysteine proteins, plasmodium, recombinant protein, structural biology, transmission-blocking, malaria

## Abstract

The 6-cysteine protein family is one of the most abundant surface antigens that are expressed throughout the *Plasmodium falciparum* life cycle. Many members of the 6-cysteine family have critical roles in parasite development across the life cycle in parasite transmission, evasion of the host immune response and host cell invasion. The common feature of the family is the 6-cysteine domain, also referred to as s48/45 domain, which is conserved across Aconoidasida. This review summarizes the current approaches for recombinant expression for 6-cysteine proteins, monoclonal antibodies against 6-cysteine proteins that block transmission and the growing collection of crystal structures that provide insights into the functional domains of this protein family.

## Introduction

### Malaria and Disease Burden

Malaria is a parasitic disease caused by the *Plasmodium* genus. Six species of malaria parasite are responsible for human disease, namely *P. falciparum*, *P. vivax*, *P. ovale curtisi, P. ovale wallikeri*, *P. malariae* and *P. knowlesi*, with *P. falciparum* responsible for most deaths. The global burden of malaria is significant, with over half of the world’s population at risk of infection. In 2020, there was an estimated 241 million cases and 627,000 deaths, with over three quarters of deaths occurring in children under five (WHO, 2021).

Having steadily declined over the last 20 years, malaria deaths increased between 2019 and 2020, reflecting the impact of the COVID-19 pandemic on services in endemic regions (WHO, 2021). In addition, resistance to anti-malarial drugs is a growing issue, with recent reports of resistance to first-line drugs in Africa (Uwimana et al., 2020; Balikagala et al., 2021; Uwimana et al., 2021), the region that accounts for approximately 95% of cases and 96% of deaths (WHO, 2021). In 2021, the WHO recommended the RTS,S/AS01 vaccine for children at risk of malaria. While this has potential for positive public health impacts, the vaccine shows a moderate efficacy of <40% (RTS S Clinical Trials Partnership, 2015). These challenges to effective malaria treatment and prevention highlight the urgency of developing effective vaccines and novel drugs to progress on-going malaria elimination programs.

**Figure 1:**
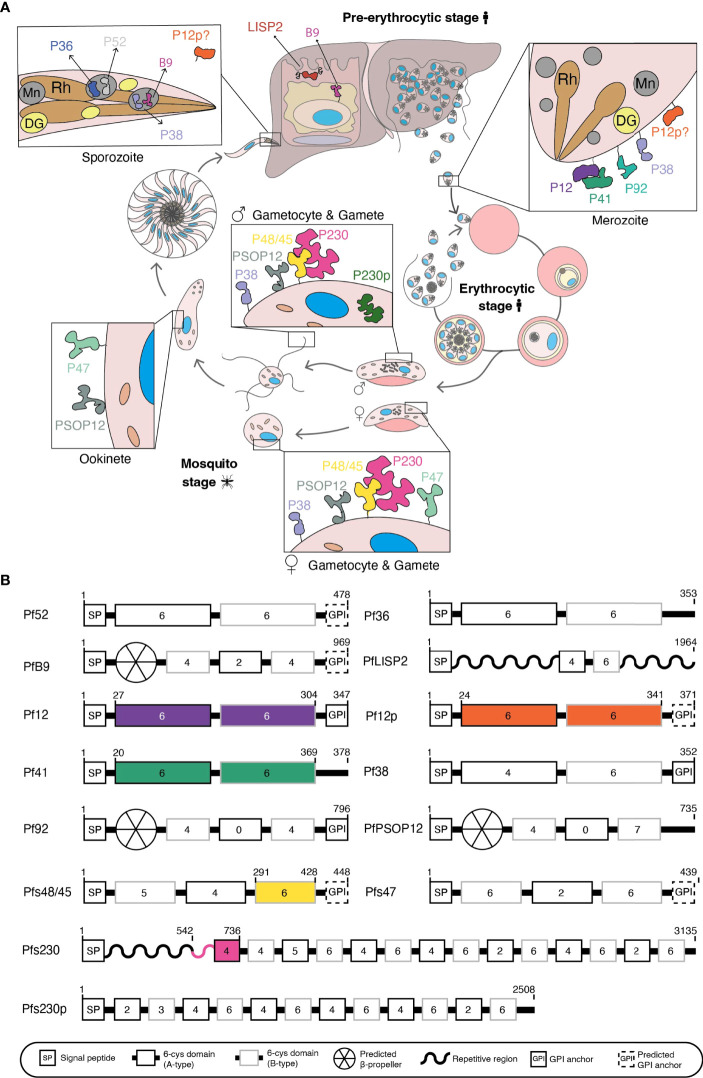
**(A)** Schematic of *Plasmodium falciparum* life cycle. 6-cysteine proteins are expressed in the pre-erythrocytic and erythrocytic stages (merozoite) of the human host and the mosquito stages (gametocyte, gametes, zygote, ookinete, and sporozoite). Mn, microneme; Rh, rhoptry; DG, dense granule. **(B)** Domain architecture of the 14 P*. falciparum* 6-cysteine proteins. The illustration shows the signal peptide (SP), the number of cysteines within each domain (number in the boxes), A-type (black box), B-type (grey box) 6-cysteine domains, validated or putative GPI-anchors, and other specific features (i.e. repetitive/non-structured region or putative β-propeller domain). Colored regions represent the part(s) of the protein for which crystal structures are available.

There has been considerable interest in 6-cysteine proteins, a family of surface-exposed and conserved *Plasmodium* proteins which are expressed throughout the parasite life cycle (**Figure 1A**) as new vaccine candidates or targets for antibody therapies that stop malaria transmission and infection.

### The 6-Cysteine Family

The first member of the 6-cysteine family to be characterized was Pfs230, a protein originally thought to be 230 kDa in size and notable for its ability to elicit antibodies that block transmission of malaria to mosquitoes (Quakyi et al., 1987). Sequencing of Pfs230 led to the identification of a cysteine-rich motif shared by Pf12 (Williamson et al., 1993) and with the increasing availability of *P. falciparum* genome sequences, this motif was subsequently identified in 14 P*. falciparum* proteins, namely Pf36, Pf52, PfLISP2, PfB9, Pf12, Pf12p, Pf41, Pf38, Pf92, Pfs48/45, Pfs230, Pfs230p, Pfs47 and PfPSOP12 (**Figure 1A**) (Templeton and Kaslow, 1999; Kappe et al., 2001; Thompson et al., 2001; Gardner et al., 2002; Gerloff et al., 2005; Arredondo et al., 2012; Orito et al., 2013; Annoura et al., 2014). These proteins are highly conserved and most have orthologues across *Plasmodium* species (Arredondo and Kappe, 2017).

The common feature of the family is the 6-cysteine domain, also referred to as the s48/45 domain (Arredondo et al., 2012; Annoura et al., 2014), which is conserved across Aconoidasida. For the purpose of this review, we will refer to this domain as the 6-cysteine domain. It is related to the SAG1-related sequence (SRS) domain that characterizes the SRS superfamily of *Toxoplasma* proteins (Arredondo et al., 2012), and both domains are likely derived from an ephrin-like host protein acquired by a common ancestor. The 6-cysteine domain folds into a β-sandwich and has up to six cysteines involved in stabilization through disulfide bonding (Templeton and Kaslow, 1999; Gerloff et al., 2005; Arredondo et al., 2012). All 6-cysteine family members have a signal sequence and between one and 14 6-cysteine domains (**Figure 1B**). In addition, some members have putative or confirmed GPI-anchors, repetitive regions, or an N-terminal β-propeller domain (**Figure 1B**).

### The 6-Cysteine Proteins in *P. falciparum*

Members of the 6-cysteine family are present across the parasite life cycle (**Figure 1A**), fulfilling many critical roles in parasite development. The life cycle of *P. falciparum* begins when an infected female *Anopheles* mosquito takes a blood meal from a human. Sporozoites are injected from the mosquito’s salivary glands into the skin and travel through the bloodstream to the liver. Within the liver, sporozoites mature into liver schizonts, which in turn rupture and release merozoites into the bloodstream. Merozoites invade red blood cells to initiate the blood stage cycle. Within 48 hours, the malaria parasite replicates into 16-36 new merozoites that proceed to invade more red blood cells. Within the bloodstream a subset of parasites differentiates to form male and female gametocytes. When a female *Anopheles* mosquito takes a blood meal, gametocytes are taken up into the mosquito midgut, where male microgametes and female macrogametes egress from red blood cells. Fertilization occurs between microgametes and macrogametes, forming zygotes. Zygotes elongate to form ookinetes, which then develop into oocysts on the mosquito midgut. Oocysts rupture and release sporozoites, which travel to the mosquito’s salivary glands, ready to infect another human during the next blood meal.

We will provide an overview of the 6-cysteine protein family on their functional diversity, respective recombinant protein expression, transmission blocking capability and structural scaffold. Their cellular localization and orthologues have been reviewed in detail previously (Arredondo and Kappe, 2017).

#### Pre-Erythrocytic Stages

Pf36, Pf52, PfLISP2 and PfB9 are expressed during the liver stages. Pf36 and Pf52 have two 6-cysteine domains, but only Pf52 contains a predicted GPI anchor. These two proteins are localized to the micronemes of salivary sporozoites and are important for parasitophorous vacuole membrane formation within hepatocytes (Arredondo et al., 2018). Pf36 is attached to the parasite membrane where it binds host receptors EphA2 (Kaushansky et al., 2015), CD81 and SR-B1 (Manzoni et al., 2017) to facilitate sporozoite invasion, although the exact mechanisms by which it is anchored to the membrane or facilitates invasion have not yet been elucidated. In *P. yoelii*, P36 forms a complex with GPI-anchored P52 and both proteins are likely involved in the formation of the liver stage parasitophorous vacuole (Arredondo et al., 2018). A genetically attenuated parasite (GAP) vaccine with deletions of Pf36, Pf52 and another liver stage antigen SAP1 has undergone a Phase I clinical trial (NCT03168854), and was able to elicit antibodies that inhibited sporozoite invasion and traversal (Kublin et al., 2017).

PfLISP2, also known as Sequestrin, has two 6-cysteine domains (Annoura et al., 2014). It is expressed in the mid to late liver stages and can be used as a marker of liver stage development (Gupta et al., 2019). PfLISP2 is exported into the host hepatocyte (Orito et al., 2013). The precise function of PfLISP2 is not clear but it may have a dual role in schizogony and egress from the liver as well as immune evasion. A GAP vaccine including a PfLISP2 deletion has been shown to protect mice from malaria infection (Vaughan et al., 2018).

PfB9 has three 6-cysteine domains and a predicted GPI anchor (**Figure 1B**). It is predicted to localize to the plasma membrane of liver stage parasites (Annoura et al., 2014) and the micronemes of sporozoites in *P. berghei* (Fernandes et al., 2021). The function of PfB9 has not been precisely defined, but knockout parasites display growth arrest in the liver and a lack of parasitophorous vacuole markers, indicating a critical role for PfB9 in liver-stage development and parasitophorous vacuole integrity (Annoura et al., 2014). A Phase I/2a trial (NCT03163121) of a GAP vaccine including a PfB9 deletion showed the vaccine to be safe and immunogenic, although the strength of protective efficacy is yet to be established (Roestenberg et al., 2020).

#### Erythrocytic Stages

Pf12, Pf12p, Pf41, Pf92 and Pf38 are expressed during the blood stage cycle. Pf12 is the structural archetype of the 6-cysteine family as well as being its smallest member (Gerloff et al., 2005). It is comprised of two 6-cysteine domains and a GPI anchor (Tonkin et al., 2013). Pf12 is expressed on the surface of schizonts and merozoites (Taechalertpaisarn et al., 2012; Tonkin et al., 2013). Pf12 forms a heterodimer with Pf41 (Taechalertpaisarn et al., 2012; Crosnier et al., 2013; Tonkin et al., 2013; Parker et al., 2015; Dietrich et al., 2022), another blood stage 6-cysteine protein with similar localization patterns (Sanders et al., 2005). Pf41 has two 6-cysteine domains and no GPI anchor, likely being tethered to the membrane *via* its interaction with Pf12. Deletions of these genes in *P. falciparum* produced no observable phenotype in blood stage parasites (Taechalertpaisarn et al., 2012) and thus the functions of these proteins remain unknown. Seroprevalence for anti-Pf12 and -Pf41 antibodies is high in malaria-exposed individuals, although this does not always correlate strongly with protection against clinical disease (Richards et al., 2013; Osier et al., 2014; Kana et al., 2018; Kana et al., 2019).

Pf12p, a paralog of Pf12, has two 6-cysteine domains and a GPI anchor. The recently solved crystal structure of Pf12p revealed its structural similarity to Pf12, despite which it does not interact with Pf41 (Dietrich et al., 2021). Transcription of P12p has been reported in the blood stages (Bozdech et al., 2003; Lopez-Barragan et al., 2011; Toenhake et al., 2018; Tang et al., 2020) and expression has been detected in sporozoites (Lasonder et al., 2008; Lindner et al., 2013) but its localization, function and precise expression patterns are unknown.

Pf92 has three 6-cysteine domains and a confirmed GPI anchor (**Figure 1B**). It is expressed in blood stage schizonts and merozoites, where it is localized to the parasite surface (Sanders et al., 2005; Gilson et al., 2006). Pf92 recruits Factor H, a host complement regulator, to the surface of merozoites as an immune evasion strategy to prevent complement-mediated lysis (Kennedy et al., 2016). Pf92 is the only member of the 6-cysteine proteins without a known orthologue in rodent *Plasmodium* species.

Pf38 has two 6-cysteine domains and a confirmed GPI anchor (Sanders et al., 2005; Gilson et al., 2006). In *P. falciparum* it has been detected on the surface (Feller et al., 2013) and apical end (Sanders et al., 2005) of the asexual blood stages, as well as on gametocytes, gametes and zygotes (Feller et al., 2013; Arredondo and Kappe, 2017). In *P. yoelii* P38 has also been localized to the micronemes of sporozoites (Harupa, 2015). The function of this protein has yet to be fully elucidated. In *P. berghei* and *P. yoelii*, P38 gene deletions have no obvious effect on asexual or sexual parasite development (van Dijk et al., 2010). However, in *P. falciparum*, Pf38-derived peptides have an inhibitory effect on red blood cell invasion (Garcia et al., 2009) and polyclonal anti-Pf38 antibodies are capable of inhibiting blood-stage growth and zygote formation (Feller et al., 2013).

#### Mosquito Stages

Pfs48/45, Pfs230, Pfs47, Pfs230p and PfPSOP12 are expressed during the sexual stages. Pfs48/45 has three 6-cysteine domains and a putative GPI-anchor and is localized to the surface of gametocytes and gametes (Vermeulen et al., 1986; Kocken et al., 1993). Knockout studies in *P. falciparum* and *P. berghei* implicate a role in male fertility, with P48/45-deficient males unable to attach to female gametes, leading to reduced ookinete production (van Dijk et al., 2001; van Dijk et al., 2010; Ramiro et al., 2015). It remains unclear, however, whether this effect is due to the loss of Pfs48/45 itself or due to an absence of Pfs230 from the parasite surface, which is anchored to the membrane by its interaction with Pfs48/45 (Eksi et al., 2006). Recognition of Pfs48/45 by human sera correlates with the ability of sera to block parasite transmission (Graves et al., 1998; Mulder et al., 1999; van der Kolk et al., 2006; Bousema et al., 2010; Ouédraogo et al., 2011; Stone et al., 2018) and antibodies against Pfs48/45 have transmission-blocking activity (Targett, 1988; Targett et al., 1990; Roeffen et al., 2001; Outchkourov et al., 2008; Lennartz et al., 2018; Stone et al., 2018). Pfs48/45-based transmission-blocking vaccines are under development against both *P. falciparum* (Theisen et al., 2017; Mistarz et al., 2017; Singh et al., 2017a; Singh et al., 2017b; Mamedov et al., 2019; Lee et al., 2020; Singh et al., 2021a) and *P. vivax* (Arevalo-Herrera et al., 2015; Tachibana et al., 2015; Cao et al., 2016; Arevalo-Herrera et al., 2021; Arevalo-Herrera et al., 2022).

Pfs230 is the largest member of the 6-cysteine family at >300 kDa and with 14 6-cysteine domains. It is expressed from stage II gametocytes until the end of fertilization and is localized to the surface of gametocytes and gametes (Rener et al., 1983; Vermeulen et al., 1985; van Dijk et al., 2010), likely *via* its interaction with GPI-anchored Pfs48/45 (Kumar, 1987; Williamson et al., 1996). In addition to this interaction, it is suggested that Pfs230 forms a multimeric protein complex involving other sexual stage antigens, Pfs25 and PfCCp family proteins (Simon et al., 2016). Pfs230 undergoes two independent N-terminal cleavage events during gametogenesis, resulting in 300 and 307 kDa versions of the protein (Williamson et al., 1996; Brooks and Williamson, 2000). Knockout studies show that Pfs230 is critical for male fertility, with Pfs230-deficient males unable to bind to red blood cells and establish exflagellation centers in *P. falciparum* (Eksi et al., 2006). In *P. berghei*, Pbs230-deficient males fail to recognize female gametes (van Dijk et al., 2010). Pfs230 has long been considered as a potential transmission-blocking malaria vaccine candidate (Quakyi et al., 1987) and seropositivity of human sera against Pfs230 is a predictor for transmission-blocking immunity (Premawansa et al., 1994; Graves et al., 1998; Mulder et al., 1999; van der Kolk et al., 2006; Bousema et al., 2010; Ouédraogo et al., 2011; Stone et al., 2018). As such, a number of recombinant N-terminal fragments of Pfs230 are under investigation as vaccine candidates (Farrance et al., 2011; Tachibana et al., 2011; MacDonald et al., 2016; Lee et al., 2017; Chan et al., 2019; Wetzel et al., 2019; Miura et al., 2019; Lee et al., 2019a; Lee et al., 2019b; Coelho et al., 2019; Huang et al., 2020; Healy et al., 2021; Coelho et al., 2021) in addition to a Pfs230-Pfs48/45 chimera (Singh et al., 2019; Singh et al., 2020; Singh et al., 2021b).

Pfs47, the paralog of Pfs48/45, is comprised of three 6-cysteine domains and a predicted GPI anchor. It is localized to the surface of female gametes and gametocytes (van Schaijk et al., 2006) and ookinetes (Molina-Cruz et al., 2013). Pfs47 binds to a mosquito receptor protein, termed P47Rec, through which it is thought to mediate evasion of the mosquito complement-like system (Molina-Cruz et al., 2013; Ramphul et al., 2015; Molina-Cruz et al., 2020). In *P. berghei*, Pbs47 serves a dual function in gamete fertility and evasion of the mosquito complement-like response (van Dijk et al., 2010; Ukegbu et al., 2017). A number of Pfs47-based vaccine candidates have been developed and shown to elicit transmission-blocking antibodies against *P. falciparum* (Canepa et al., 2018; Yenkoidiok-Douti et al., 2019; Yenkoidiok-Douti et al., 2021) and *P. berghei* (Yenkoidiok-Douti et al., 2020).

Pfs230p, the paralog of Pfs230, contains 12 6-cysteine domains. Pfs230p is expressed only in male gametocytes (stages IV-V) and is the only 6-cysteine protein known to be localized to the cytoplasm in *P. falciparum* (Eksi and Williamson, 2002; van Dijk et al., 2010; Schneider et al., 2015). A critical role for P230p in ookinete formation has been described in *P. falciparum*, although this function is not observed in rodent malaria parasites (van Dijk et al., 2010; Marin-Mogollon et al., 2018).

PfPSOP12 has three predicted 6-cysteine domains (**Figure 1B**). It is expressed in gametocytes, ookinetes and oocyts and is localized to the surface of the parasite (Sala et al., 2015), although this protein has no predicted GPI anchor (**Figure 1B**). Knockout studies in *P. berghei* have demonstrated a mild effect on oocyst production and anti-PbPSOP12 antibodies have transmission-blocking activity (Ecker et al., 2008; Sala et al., 2015), both suggesting a potential role in fertility. In contrast, the function of *P. falciparum* PfPSOP12 has yet to be characterized.

The 6-cysteine proteins act throughout the malaria life and some have critical roles in parasite development. However, the precise function, structure and transmission-blocking potential of many members have not been fully elucidated. Below we describe methods that have been used to produce and characterize this protein family thus far and discuss methods that could be used to further understand the interactions of the 6-cysteine proteins and target them effectively.

## Expression of Recombinant 6-Cysteine Proteins

Recombinant protein expression of properly folded 6-cysteine proteins is important for the structural and biochemical characterization of this protein family and the generation of specific antibodies. However, there are significant challenges for expressing sufficient yields of correctly folded protein. In prokaryotic expression systems, additional components such as conjugation to a fusion partner or co-expression of folding catalysts are often required for proper disulfide bonding of the 6-cysteine domain. In eukaryotic systems, glycans on *Plasmodium* proteins are truncated compared to those of other eukaryotes (Bushkin et al., 2010; Swearingen et al., 2016) and incorrect glycosylation can affect the conformation of proteins expressed in these systems. Enzymatic deglycosylation or mutation of glycosylation sites can be employed to prevent erroneous glycosylation, although glycosylation patterns may still differ to the native protein. Due to the AT richness of the *Plasmodium* genome, codon optimization is often required for optimum yields between the different recombinant expression systems. Below and in **Supplementary Table 1** we summarize the various protein expression systems used to produce recombinant 6-cysteine proteins and the strategies employed to induce correct conformation.

### Prokaryotic Systems for the Expression of 6-Cysteine Proteins

#### *Escherichia coli* Expression

Initial attempts to express recombinant 6-cysteine proteins were carried out in *E. coli* (Kocken et al., 1993; Williamson et al., 1993; Riley et al., 1995; Williamson et al., 1995). However, correct disulfide bond formation can be difficult to achieve in this system as protein production occurs largely in the cytoplasm, while disulfide bond formation is carried out by oxidoreductases in the periplasm. For this reason, early attempts to produce full length Pfs48/45 resulted in incorrectly folded protein unable to elicit transmission-blocking antibodies in mice or rabbits (Milek et al., 1998).

Solubility tags such as glutathione-*S*-transferase (GST) or maltose binding protein (MBP) fused to the recombinant protein can assist folding. MBP was used for the early expression of Pfs230 fragments (Williamson et al., 1993; Riley et al., 1995; Williamson et al., 1995; Milek et al., 1998; Bustamante et al., 2000) and GST for Pfs48/45 fragments (Kocken et al., 1993; Milek et al., 1998; Outchkourov et al., 2007). Co-expression of an MBP-tagged Pfs48/45 fragment (10C, aa 159-428) with periplasmic folding catalysts produced a properly folded protein with an increased yield relative to the GST fusion of the same fragment (Outchkourov et al., 2007; Outchkourov et al., 2008). The full ectodomain and single domain constructs of Pfs47 have been expressed as a fusion to *E. coli* protein thioredoxin. These proteins were used to identify domain 2 (D2) as the region of Pfs47 targeted by transmission-blocking antibodies (Canepa et al., 2018) and to elucidate the essential function of Pbs47 (Ukegbu et al., 2017). A fusion of the Pvs48/45 ectodomain to thioredoxin was recognized by sera from naturally infected individuals and elicited transmission-blocking antibodies (Arevalo-Herrera et al., 2015). A fusion of Pfs48/45 10C with the granule lattice protein of *Tetrahymena thermophila* similarly yielded a correctly folded protein that elicited transmission-blocking antibodies (Agrawal et al., 2019).

Other strategies to encourage correct folding without reliance on a fusion partner include the use of *E. coli* strains that enhance disulfide bond formation in the cytoplasm, which was used to investigate the immunogenicity and immunoreactivity of Pvs48/45 (Arevalo-Herrera et al., 2015; Arevalo-Herrera et al., 2021; Arevalo-Herrera et al., 2022). Correctly folded full-length Pfs48/45 has also been reported without a fusion partner through codon harmonization (Chowdhury et al., 2009).

Despite the challenges, *E. coli* remains a popular expression system and has recently been used for pre-clinical evaluation of vaccine candidates Pfs47 (Yenkoidiok-Douti et al., 2019; Yenkoidiok-Douti et al., 2021), Pbs47 (Yenkoidiok-Douti et al., 2020) and Pfs48/45 (Pritsch et al., 2016) in animal immunization studies. The system has been used to produce Pf12 D2 for structural characterization (Arredondo et al., 2012) and the full ectodomains of Pfs48/45 and Pvs48/45 to demonstrate the cross-reactivity of immune responses to these antigens (Cao et al., 2016).

#### *Lactococcus lactis* Expression

The *L. lactis* system has been used to express Pf12, Pf38 and Pf41 (Singh et al., 2018; Kana et al., 2018) in addition to Pfs48/45- and Pfs230-based vaccine candidates. This system can be optimized to produce correctly folded protein with yields of 25 mg/L, which is sufficient for use in clinical studies (Singhet al., 2017a; Singh et al., 2021).

Vaccine candidates based on a fusion of a P48/45 fragment with the R0 region of glutamate-rich protein (GLURP) have been expressed in *L. lactis.* Fusion of the Pfs48/45 10C fragment to R0 yielded a correctly folded protein that elicited transmission-blocking antibodies (Theisen et al., 2014; Roeffen et al., 2015) and it is hypothesized that fusion to R0 stabilizes Pfs48/45 and enhances expression. A vaccine candidate comprised of R0 and the Pfs48/45 6C fragment (aa 291-428) is also capable of eliciting transmission-blocking antibodies (Singh et al., 2015; Singh et al., 2017a), and protocols have been developed for expression of this construct as a virus-like particle (VLP) (S.K. Singh et al., 2017b) and under cGMP settings (Singh et al., 2021a). A fusion of Pfs48/45 6C to R0 and a region of merozoite surface protein 3 (GMZ2.6C) elicited parasite-specific antibodies (Baldwin et al., 2016) and a 10C version of this vaccine candidate (GMZ2’.10C) elicited transmission-blocking antibodies (Mistarz et al., 2017). A fusion protein comprising Pfs48/45 6C and the pro-domain of Pfs230 (aa 443-590) is under investigation as a vaccine candidate, with the glutamate-rich pro-domain assisting the proper folding of Pfs48/45 in a similar manner to the R0 region of GLURP (Singh et al., 2019; Singh et al., 2021b).

Successful expression of Pfs48/45 6C and a region of Pfs230 (aa 443-590) in *L. lactis* without fusion partners has also been reported, enabling investigation of the immune response against these 6-cysteine proteins without needing to account for the immune response generated by the fusion partner (Acquah et al., 2017).

### Eukaryotic Systems for the Expression of 6-Cysteine Proteins

#### Baculovirus/Insect Cell Expression

The baculovirus/insect cell system was used to express Pfs48/45 in the first reported expression of a 6-cysteine protein (Kocken et al., 1993) and has since been employed for investigation of 6-cysteine protein structure, interactions and immunogenicity.

Expression of recombinant Pf12 (Tonkin et al., 2013), Pf12p (Dietrich et al., 2021) and Pf41 (Parker et al., 2015) *via* this system has yielded high resolution crystal structures of these proteins, as well as the Pf12-Pf41 complex (Dietrich et al., 2022). Recombinant Pfs47 expressed using the baculovirus system was used to identify its binding partner, the mosquito receptor protein P47Rec, and elucidate the involvement of this interaction in evading the mosquito immune system (Molina-Cruz et al., 2020).

The baculovirus system also produced full-length Pfs48/45 and Pfs48/45 6C vaccine candidates, though only the latter elicited transmission-reducing antibodies (Lee et al., 2020). A PbPSOP12 vaccine candidate produced in this system showed modest transmission-reducing activity (Sala et al., 2015). Pfs230C1 (aa 443-731) produced in this system was able to elicit antibodies that hinder parasite development (Lee et al., 2017; Miura et al., 2019; Huang et al., 2020). Optimization of the production of Pfs230C1 resulted in a final yield of 10 mg/L with the potential to produce over 1g of protein (Lee et al., 2019a). Expression of a shorter Pfs230 fragment, Pfs230D1+ (aa 552-731), also elicited antibodies with transmission-blocking activity and yields were two-fold higher than obtained for Pfs230C1 at 23 mg/L (Lee et al., 2019b).

#### Mammalian Cell Expression

Recombinant proteins expressed in HEK293 cells were used to elucidate the interactions of Pf12 and Pf41 (Taechalertpaisarn et al., 2012) and of *P. yoelii* P36, which in complex with P52 engages host receptor EphA2 (Kaushansky et al., 2015). Pfs48/45 6C was expressed in this system for crystallization in complex with transmission-blocking mAb 85RF45.1 (Kundu et al., 2018). The Chinese Hamster Ovary (CHO) cell line was used to express full-length Pvs48/45, which demonstrated higher immunogenicity than when expressed in *E. coli* (Arevalo-Herrera et al., 2021; Arevalo-Herrera et al., 2022).

The scalability of mammalian cell expression was utilized to produce a library of recombinant proteins including P12, P38, P41 and P92 from *P. falciparum*, *P. vivax, P. malariae, P. ovale and P. knowlesi*. The library was used to investigate cross-reactivity of sera across *Plasmodium* as a serological assay for diagnosing exposure to *P. ovale, P. malariae* or *P. knowlesi* (Muller-Sienerth et al., 2020). In *P. falciparum*, a library of secreted and surface merozoite proteins including Pf12, Pf12p, Pf38, Pf92 and Pf41 was produced. The library was used to confirm the interaction of Pf12 with Pf41 and demonstrate that these 6-cysteine proteins, with the exception of Pf12p, are immunoreactive with immune sera (Crosnier et al., 2013). Similarly, a recombinant protein library of *P. vivax* antigens confirmed the interaction of Pv12 with Pv41 and identified additional potential interaction partners for Pv12 (Hostetler et al., 2015).

#### Plant-Based Expression

The Australasian tobacco plant *Nicotiana benthamiana* has been used for the expression of recombinant 6-cysteine proteins including Pf38, which was recognized by sera of semi-immune donors and elicited antibodies with transmission-reducing activity upon immunization of mice, although only a low yield of 4 mg/kg of fresh leaf biomass was obtained (Feller et al., 2013). In contrast, expression of a Pfs230 region referred to as 230CMB (aa 444-730) in *N. benthamiana* resulted in yields of 800 mg/kg of fresh whole leaf tissue (Farrance et al., 2011). Antibodies generated against this recombinant protein in rabbits were able to bind native parasite and demonstrated transmission-blocking activity with a reduction of >99% in oocyst counts (Farrance et al., 2011).

In this system, *in vivo* deglycosylation of 6-cysteine proteins has been explored through co-expressing 6-cysteine proteins with enzymes PNGase F or Endo H. Co-expression of a region of Pfs48/45 (aa 28-401) referred to as Pfs48F1, which contains seven putative N-glycosylation sites, and PNGase F to remove all N-linked glycans resulted in a yield of 50 mg/kg of fresh leaf biomass. Anti-Pfs48/45 monoclonal antibodies (mAbs) showed higher affinity to the *in vivo* deglycosylated protein compared to the glycosylated protein and slightly higher affinity to the protein deglycosylated *in vivo* compared to *in vitro* (Mamedov et al., 2012). Developments to this protocol have included expressing Pfs48F1 and PNGase F from a single vector (Prokhnevsky et al., 2015) and co-expressing full length Pfs48/45 (aa 28-428) or Pfs48/45 10C with Endo H, which removes only certain types of N-linked carbohydrates. This resulted in a higher yield of protein than co-expression with PNGase F (52 mg/kg *vs* 27 mg/kg, respectively) that was more stable and elicited antibodies with stronger transmission-reducing activity (Mamedov et al., 2017; Mamedov et al., 2019).

#### Yeast-Based Expression

Expression of Pfs48/45 in *Saccharomyces cerevisiae* was initially attempted but did not produce sufficient levels of protein for detection. In contrast, recombinant Pfs48/45 could be detected in *P. pastoris*, but with an expression efficiency of only 1% (Milek et al., 2000). More recently, efficient expression of Pfs230D1M (aa 542-736) was achieved in *P. pastoris* for use in a vaccine currently undergoing Phase I and II clinical trials (MacDonald et al., 2016; Coelho et al., 2019; Healy et al., 2021). This system has produced recombinant Pfs230D1 for crystallization with transmission-blocking antibodies LMIV230–01 (Coelho et al., 2021) and 4F12 (Singh et al., 2020). *H. polymorpha* has been employed to express Pfs230 constructs in a VLP alongside sexual stage antigen Pfs25, which elicited antibodies with transmission-reducing activity (Chan et al., 2019; Wetzel et al., 2019).

#### Other Eukaryotic Expression Systems

The stable Drosophila Schneider-2 cell line was employed to express full-length Pfs48/45 due to its ability to produce large quantities of correctly folded protein without the need for a fusion partner or carrier protein. The recombinant protein was recognized by known anti-P48/45 mAbs and induced antibodies with transmission-blocking activity in mice, suggesting it had adopted the correct conformation (Lennartz et al., 2018). A Pfs48/45 6C fragment produced by the same method was crystallized in complex with transmission-blocking antibody 85RF45.1, representing one of the first crystal structures of a Pfs48/45 fragment (Lennartz et al., 2018).

*Chlamydomonas reinhardtii*, a species of green algae, has been investigated as a low-cost option for production of vaccine candidate Pfs48/45. A region comprising aa 178-448 was successfully expressed and could be recognized by the mAb IIC5-10, known to recognize Pfs48/45, indicating the protein folded in the correct conformation (Jones et al., 2013).

### Cell Free Systems

Cell free systems such as the wheat germ cell-free (WGCF) system offer the potential for rapid, high-throughput protein expression of *Plasmodium* proteins (Tsuboi et al., 2008). For this reason, cell free systems have been used to express multiple antigens for high-throughput screening of patient sera against an array of antigens. Such studies have shown that antibodies against Pf38 have an intermediate association with protection against symptomatic malaria (Richards et al., 2013) and antibody levels against Pfs230C may be associated with age (Muthui et al., 2021). Unfortunately, not all antigens appear to be amenable to expression *via* this system, with production of recombinant Pfs48/45, Pfs47 and PfPSOP12 proving unsuccessful (Muthui et al., 2021). This system has also been used to produce multiple vaccine candidates for functional comparison, supporting the further development of Pfs230C as a vaccine candidate (Miura et al., 2013).

The WGCF system was used to express multiple fragments of Pfs230, which due to its size is difficult to express as a full-length protein. Expression of fragments spanning the Pfs230C region (aa 443-1132) demonstrated that truncated fragments were capable of eliciting transmission-blocking antibodies (Tachibana et al., 2011). This contrasts observations for *E. coli* where only the full Pfs230C fragment was capable of eliciting transmission-blocking antibodies (Bustamante et al., 2000), suggesting the native topology of the proteins was better retained by the WGCF-produced fragments. Expression of protein fragments that together span the entirety of Pfs230 was used to further pinpoint the functional transmission-blocking epitopes of Pfs230 (Tachibana et al., 2019; Miura et al., 2022).

Cell free systems have been used to express *P. vivax* orthologues Pv12 (Li et al., 2012) and Pv41 (Cheng et al., 2013), demonstrating the immunoreactivity of naturally acquired sera with these antigens. The recombinant proteins were also used to generate anti-Pv12 and anti-Pv41 antibodies used to reveal the subcellular localization of these proteins. High-throughput screening of immune sera against *P. vivax* proteins identified 18 highly immunoreactive proteins, with Pv12 and Pv41 being among them (Chen et al., 2010). A panel of 20 P*. vivax* proteins including Pv12 were expressed by nucleic acid programmable protein array/*in vitro* transcription/translation, confirming the interaction of Pv12 with Pv41 as well as identifying additional putative interaction partners (Arevalo-Pinzon et al., 2018).

Recombinant protein expression is a bottleneck for the structural and biochemical characterization of 6-cysteine proteins. Differences between the cellular machinery of *Plasmodium* and the expression system often requires additional measures such as codon harmonisation, conjugation to a fusion partner and deglycosylation to produce properly folded protein. The choice of an appropriate expression system will depend on the protein and domain being expressed, considering its size, the numbers of disulphide bonds and glycosylation sites it contains and its intended downstream applications.

## Monoclonal Antibodies for the Characterization of 6-Cysteine Proteins

Monoclonal antibodies generated against the 6-cysteine proteins have been used to elucidate protein structure and functional domains, and some are capable of inhibiting parasite development and transmission. In this review, we will focus on the description of these inhibitory mAbs against the three transmission-blocking vaccine candidates, Pfs230, Pfs48/45, and Pfs47 (**Supplementary Table 2**).

### Pfs230

The mature form of Pfs230 contains an additional non-structured pro-domain region of ˜100 amino acids upstream of the first 6-cysteine domain (D1) (Carter et al., 1995; Brooks and Williamson, 2000; Gerloff et al., 2005). Epitope mapping of anti*-*Pfs230 antibodies suggests that part of the pro-domain and D1 of Pfs230 can elicit transmission-blocking antibodies (Tachibana et al., 2019; Miura et al., 2022) (**Supplementary Table 2**).

To date, 20 inhibitory mAbs specific for *P. falciparum* Pfs230 have been reported (**Supplementary Table 2**), which reduce the formation of oocysts on the mosquito midgut to varying degrees (42.2%-100%) when assayed using the standard membrane feeding assay (SMFA) (**Supplementary Table 2**). Of these antibodies, 16 (63F2A2.2a & 2b, 2B4, 1B3, 11E3, 12F10, 1H2, 3G9, 7A6, 8C11, 17E9, 4C10, 11C12F7, 21C1, 12A1A5, and 1A3-B8) were generated *via* direct animal immunization with the sexual stage parasites (either intact cells or whole cell lysate) (Rener et al., 1983; Quakyi et al., 1987; Read et al., 1994; Roeffen et al., 1995a; Roeffen et al., 1995; Williamson et al., 1995). Two mAbs, 4F12 and 5H1, were obtained *via* animal immunization with recombinant protein (MacDonald et al., 2016; Singh et al., 2020), and two mAbs, LMIV230-01 and LMIV230-02, were directly isolated from memory B cells of vaccinated Malian adults (Coelho et al., 2019). In these studies, recombinant Pfs230 proteins containing parts of the pro-domain region and D1 of Pfs230 were used as antigens (MacDonald et al., 2016; Coelho et al., 2019; Singh et al., 2020). Using structural biology approaches, the 4F12 and LMIV230-01 binding epitopes were identified to be within conserved regions of Pfs230 D1 (Coelho et al., 2019; Singh et al., 2020). Three additional transmission-blocking mAbs (1A3-B8, 11C5-B10, and 29F432) are reactive against both Pfs230 and Pfs48/45 (Rener et al., 1983; Quakyi et al., 1987; Read et al., 1994; Roeffen et al., 1995a; Roeffenet al., 1995b; Williamson et al., 1995).

The inhibitory mechanism of anti-Pfs230 antibodies has been thought to be predominantly complement-dependent (Healer et al., 1997), which could explain the higher transmission reducing activity observed for Pfs230 in a Phase I clinical trial compared to benchmark transmission-blocking vaccine candidate Pfs25 (Healy et al., 2021). Indeed, many Pfs230 mAbs lose their transmission-blocking ability in the absence of human serum (**Supplementary Table 2**). The chimeric rh4F12 mAb, containing the original mouse fragment antigen binding (Fab) region fused with human IgG1 crystallizable fragment (Fc), showed increased potency relative to the original mouse antibody, possibly due the presence of human IgG1 Fc, which is thought to enhance complement fixing activity (Singh et al., 2020). However, 4F12, LMIV230-01 and 1A3-B8 can inhibit transmission in the absence of complement activity (Rener et al., 1983; MacDonald et al., 2016; Singh et al., 2020; Coelho et al., 2021) (**Supplementary Table 2**), suggesting that complement-independent inhibitory mechanisms exist for anti-Pfs230 antibodies.

### Pfs48/45

Anti-Pfs48/45 antibodies with transmission-blocking activity target at least four epitope groups (epitopes I, IIb, III, and V) that span all three 6-cysteine domains of Pfs48/45 (N. Targett, 1988; Targett et al., 1990; Roeffen et al., 2001b; Outchkourov et al., 2007; Outchkourov et al., 2008; Lennartz et al., 2018)(**Supplementary Table 2**). The disulfide bonds within the central and C-terminal 6-cysteine domains are critical for the presentation of the transmission-blocking epitopes, but dispensable for epitope presentation on the N-terminal domain of Pfs48/45 (Outchkourov et al., 2007).

There are 15 reported transmission-blocking anti-Pfs48/45 mAbs, with relatively well characterized epitopes. When assessed by SMFA, all 15 mAbs were able to reduce oocyst formation by 55.5%-100% under the conditions tested (**Supplementary Table 2**). Six mAbs, 85RF45.1 and its humanized version TB31F, 85RF45.5, 32F3, 32F5, and 3E12, were generated by animal immunization with intact sexual stage parasites (Vermeulen et al., 1985; Targett, 1988; Carter et al., 1990; Targett et al., 1990; Roeffen et al., 2001a). All these antibodies recognize the C-terminal domain of Pfs48/45 except for 85RF45.5, which recognizes the N-terminal domain. The remaining eight antibodies were generated *via* animal immunization with full-length Pfs48/45 protein obtained either through affinity purification from *P. falciparum* cell lysate (82C4-A9, 81D3-D2, 42A6-F3, 84A2-A4, and 82D6-A10) (Targett, 1988), or recombinant protein expression (1F10, 3G3, 6A10, 10D8) (Lennartz et al., 2018). These eight antibodies, including two unique IgM antibodies 82C4-A9 and 81D3-D2, recognize either the N-terminal or central domains of Pfs48/45 (Targett, 1988; Lennartz et al., 2018).

The most potent anti-Pfs48/45 mAb is the humanized version of mAb 85RF45.1 (TB31F), which reduces oocyst formation in mosquitos by 80% at a concentration of 1-2 μg/mL (Roeffen et al., 2001a; Kundu et al., 2018), approximately 15x more potent than the anti-Pfs25 mAb 4B7 (IC_80_ of 30.7 μg/mL) (de Jong et al., 2021). The crystal structures of mAb 85RF45.1 and its humanized version TB31F in complex with recombinant C-terminal domain of Pfs48/45 (Pfs48/45-6C) suggest that the antibody binds to a relatively conserved conformational epitope (Kundu et al., 2018; Lennartz et al., 2018) and existing field polymorphisms do not alter antibody binding and transmission-reducing efficacy substantially (Kundu et al., 2018; Lennartz et al., 2018; Stone et al., 2018; de Jong et al., 2021) (**Supplementary Table 2**). In contrast to Pfs230, the inhibitory mechanism of anti-Pfs48/45 antibodies are complement-independent (Patel and Tolia, 2021).

### Pfs47

Recent studies show that immunization with D2 of Pfs47 and Pbs47 elicited antibodies with transmission-blocking activity (Canepa et al., 2018; Yenkoidiok-Douti et al., 2020), whereas antibodies that preferentially bind D1 and D3 did not exhibit transmission-blocking activity (Canepa et al., 2018; Yenkoidiok-Douti et al., 2020). Pfs47 D2-specific inhibitory mAbs, IB2 and BM2, have been shown to bind to the linear epitope on the central region of D2 and reduce transmission by >85% at 200 μg/mL. In contrast, JH11, which binds the N-terminal region of D2, increases transmission (Canepa et al., 2018). The transmission-blocking activity of the Pfs47 mAbs is complement-independent like mAbs to its paralogue Pfs48/45 (Canepa et al., 2018; Yenkoidiok-Douti et al., 2020; Patel and Tolia, 2021) (**Supplementary Table 2**).

## Structural Characterization of 6-Cysteine Proteins

### The 6-Cysteine Domain

**Figure 2:**
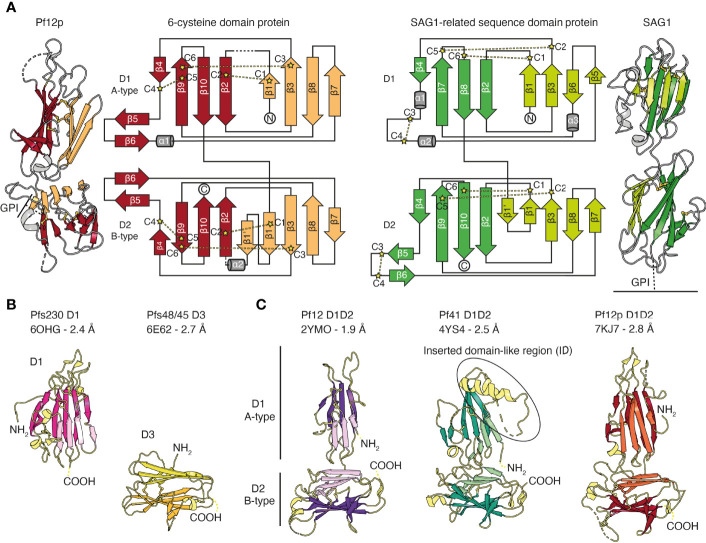
The structural scaffold of 6-cysteine domains. **(A)** Schematic representation of 6-cysteine protein Pf12p with its N-terminal A-type 6-cysteine domain (D1) and C-terminal B-type 6-cysteine domain (D2). The β-strands of the top β-sheet of the β-sandwich are colored orange and the bottom β-sheet are colored red. Cysteines forming disulfide bonds are shown in ball-and-stick representation in yellow (PDB ID 7KJ7). Dotted lines indicate unmodeled regions. Topology diagram of Pf12p colored similarly to the schematic representation. Disulfide bond connectivity is indicated by yellow lines. Topology diagram and schematic representation of SAG1 from *Toxoplasma gondii*, the prototypic member of the SRS-superfamily (right, PDB ID 1KZQ). The β-strands of the top β-sheet of the β-sandwich are colored light green and the bottom β-sheet are colored green. **(B)** Crystal structure of single 6-cysteine domains of Pfs230 D1 (left) and Pfs48/45 D3 (right). **(C)** Crystal structures of 6-cysteine proteins containing a tandem pair of A- and B-type 6-cysteine domains. PDB IDs with corresponding resolution, N-terminal domain 1 (D1), C-terminal domain 2 (D2) as well as N- and C-termini are indicated (NH2 and COOH, respectively).

The common structural feature of the family is the 6-cysteine domain. The 6-cysteine domain folds into a β-sandwich of parallel and antiparallel β-strands and contains up to six cysteines that form disulfide bonds, where cysteines C1–C2, C3–C6, C4–C5 are connected (**Figure 2A**) (Gerloff et al., 2005; Arredondo et al., 2012). ‘Degenerate’ domains containing less than six cysteines have been identified (Templeton and Kaslow, 1999). The β-sandwich is formed by two β-sheets and is usually stabilized by two disulfide bonds. A third disulfide bond connects a loop region to the core structure. Typically, a small β-sheet of two antiparallel β-strands runs perpendicular along the side of the β-sandwich. (**Figure 2A**) (Arredondo et al., 2012; Tonkin et al., 2013; Parker et al., 2015; Dietrich et al., 2021). 6-cysteine domains are present in each member, with the number of domains ranging from 2-14, and are often found in tandem pairs of A- and B-type 6-cysteine domains (**Figure 1B** and **Figure 2A**) (Carter et al., 1995; Gerloff et al., 2005). In comparison to A-type domains, the first β-strand in B-type domains is split into two parallel β-strands (β1 and β1’ in **Figure 2A**). Compared with SAG1, the prototypic member of the SRS-superfamily the 6-cysteine domains share a structural scaffold. However, differences in β-strand topology and disulfide bond connectivity exist between 6-cysteine and SRS domains (Gerloff et al., 2005; Arredondo et al., 2012) (**Figure 2A**). Tandem domains of SRS-proteins characterized to date adopt a linear head-to-tail orientation with limited D1-D2 interdomain contacts, which suggests potential mobility between SRS-domains (**Figure 2A**) (He et al., 2002; Crawford et al., 2009; Crawford et al., 2010). In contrast, tandem domains of 6-cysteine domains have a non-linear organization, potentially with restricted mobility (Tonkin et al., 2013; Parker et al., 2015; Dietrich et al., 2021).

**Figure 3:**
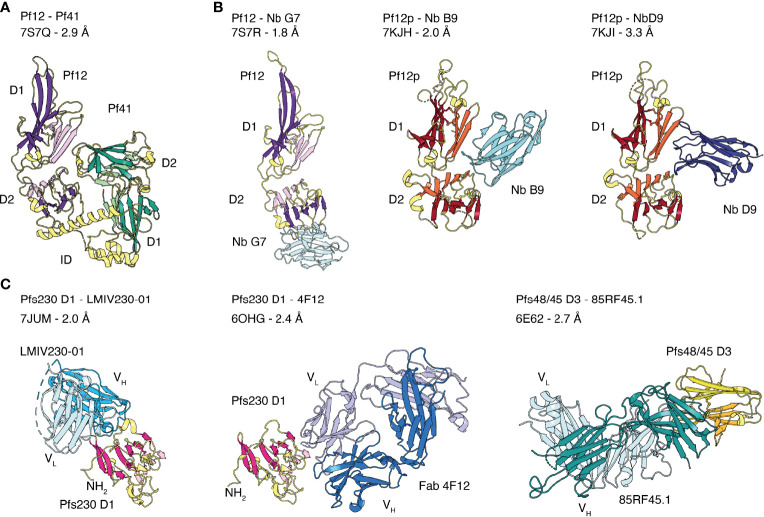
Crystal structures of the 6-cysteine proteins of *P. falciparum* in complex with another 6-cysteine protein or antibody fragments. **(A)** Crystal structure of the hetero-dimeric complex of Pf12 and Pf41 with indicated D1 and D2 domains and the inserted domain-like region **(ID)** of Pf41. **(B)** 6-cysteine proteins bound to nanobodies. Pf12 bound to Nb G7 (left), Pf12p bound to Nb B9 (middle) and Nb D9 (right). **(C)** 6-cysteine protein domains bound to Fab and single-chain fragment variable (scFv) regions. Pfs230 D1 in complex with scFv of LMIV230-01 (left) and Fab of 4F12 (middle). Pfs48/45 D3 bound to Fab of 85RF45.1 (right).

Of the 14 members of the 6-cysteine protein family, structural information is available for five *P. falciparum* proteins, namely Pf12, Pf12p, Pf41, Pfs48/45 and Pfs230 (**Figures 2B, C** and **Figure 3**). The 6-cysteine domain was first described in 2012 by the nuclear magnetic resonance (NMR) structure of the C-terminal Pf12 D2 domain, which confirmed the structural similarities with the *Toxoplasma* SRS domain (Arredondo et al., 2012). Crystal structures with single 6-cysteine domains are available for Pfs48/45 and Pfs230, which have been characterized in the presence of antibody fragments (**Figure 2B** and **Figure 3C**) (Kundu et al., 2018; Lennartz et al., 2018; Singh et al., 2020; Coelho et al., 2021). The Pf12 D2 and the Pfs48/45 D3 domains are B-type 6-cysteine domains containing three disulfide bonds. Pfs230 D1 is a degenerate 6-cysteine domain in which two disulfide bonds pin the β-sandwich together. In comparison to other 6-cysteine domains, Pfs230 D1 contains an N-terminal extension (residues 557-579) that packs against the 6-cysteine domain core (Coelho et al., 2021).

Crystal structures of the tandem domains of Pf12, Pf12p and Pf41 have been determined (**Figure 2C**) (Tonkin et al., 2013; Parker et al., 2015; Dietrich et al., 2021). Their overall structural architecture is similar in that the two 6-cysteine domains are connected by a short linker and tilted against each other. The domain-domain organization appears rather rigid as networks of interdomain contacts bury extended surface areas between 461-911 Å^2^ in these crystal structures. The domain-domain contacts are predominantly formed between residues of β-strands connecting loops of D1 and residues of the top β-sheet of D2. Two nanobodies, Nb B9 and Nb D9, were successfully crystallized in complex with recombinant Pf12p and both bind specifically to the Pf12p interdomain (D1-D2 junction) area (**Figure 3B**) (Dietrich et al., 2021). These Pf12p nanobodies were highly specific, showing very little cross-reactivity against Pf12 or Pf41, despite these proteins adopting the same two 6-cysteine domain arrangement (Dietrich et al., 2021). While the overall fold and the spatial position of the disulfide bonds are similar in Pf12, Pf41 and Pf12p, their amino acid sequence identity is low (18-27%), and loops connecting β-strands vary especially in length and conformation. In the case of Pf41, a ~110 amino acid insertion, termed the inserted domain-like region (ID), connects the last two β-strands of the D1 domain and is critical for the interaction with Pf12 (Parker et al., 2015; Dietrich et al., 2022).

Several members of the 6-cysteine protein family form hetero-complexes, such as Pfs230 and Pfs48/45, Pf12 and Pf41, and Pf36 and Pf52 (Kumar, 1987; Kumar and Wizel, 1992; Taechalertpaisarn et al., 2012; Parker et al., 2015; Arredondo et al., 2018). Recently, the first crystal structure of a 6-cysteine hetero-complex of Pf12 and Pf41 was determined (**Figure 3A**) (Dietrich et al., 2022). This Pf12-Pf41 structure identified two distinct binding sites and showed that the ID of Pf41 forms a 25 amino acid-long α-helix that binds to a concave surface of the Pf12 D2 domain. The second interaction site involves extended loops on one side of the Pf41 D2 domain that recognize residues at the Pf12 D1-D2 domain junction. Critical residues for complex formation have been identified on both proteins, suggesting that both binding sites are important for the interaction of Pf12 and Pf41. In addition, Pf12 specific nanobodies were able to inhibit complex formation between Pf12 and Pf41 (Dietrich et al., 2022). The crystal structure of one of these nanobodies, Nb G7, in complex with recombinant Pf12 showed that Nb G7 bound to a hydrophobic groove on Pf12 that overlaps with the Pf41 ID binding site (**Figure 3B**). However, Nb G7 was not inhibitory to merozoite invasion or egress (Dietrich et al., 2022), which is consistent with the genetic knockout of these proteins being dispensable for blood stage growth (Taechalertpaisarn et al., 2012).

Structures are available for Pf12 (PDB IDs 2YMO, 7S7R and 7S7Q), Pf41 (PDB IDs 4YS4 and 7S7Q) and Pfs48/45 (PDB IDs 6E62 and 6H5N) that were solved using different protein constructs that vary in sequence, amino acid range, or recombinant protein expression system (**Supplementary Table 1**). These structures agree with each other, and all described 6-cysteine domains show their predicted number of disulfide bonds.

### 6-Cysteine Protein Family Members in Complex With Inhibitory Antibody Fragments

The Fab fragment of 4F12 and the single chain fragment variable (scFv) of LMIV230-01 were crystallized in complex with Pfs230 D1 (Singh et al., 2020; Coelho et al., 2021). Both antibody fragments recognize distinct conformational epitopes on different sides of the 6-cysteine domain (**Figure 3C**). 4F12 contacts Pfs230 D1 along one edge of the β-sandwich, interacting with residues of both β-sheets and several residues of the β-strand connecting loop formed by F595-K607. The light chain of 4F12 forms more contacts with Pfs230 D1 than the heavy chain. Both, light and heavy chain together bury an average surface area of about 1500 Å^2^ on Pfs230 D1 (Singh et al., 2020). Binding of LMIV230-01 involves all six CDRs and buries a surface area of 1047 Å^2^ on Pfs230 D1 with heavy and light chains contributing to 750 Å^2^ and 297 Å^2^, respectively (Coelho et al., 2021). LMIV230-01 contacts residues of five β-strands all located on one side of the β-sandwich, a β-strand connecting loop and residues of the long N-terminal extension of Pfs230 D1. The interaction of LMIV230-01 and Pfs230 D1 is mostly stabilized by hydrophobic contacts, and five residues of Pfs230 are involved in hydrogen bonds or salt bridges with LMIV230-01. The epitope is conserved as all major polymorphisms identified from 2512 analyzed sequences from Africa and Asia are outside the binding site of LMIV230-01.

MAb 85RF45.1 is a potent transmission-reducing antibody targeting the C-terminal D3 domain of Pfs48/45 (Roeffen et al., 2001b; Kundu et al., 2018; Lennartz et al., 2018). There are two crystal structures of the Fab fragment of 85RF45.1 in complex with the D3 domain of Pfs48/45 at 2.7 Å (Kundu et al., 2018) and 3.2 Å (Lennartz et al., 2018) resolution. These two crystal structures, Protein Data Bank (PDB) entry 6E62 and 6H5N, align well with root mean square deviations (r.m.s.d.) of ~0.977 Å. The r.m.s.d. of two superimposed protein structures describes the average distance between corresponding atom positions. The smaller the r.m.s.d. value the more similar the two structures are, where a value of zero means a perfect fit. Here, we will describe the interactions between the Fab of 85RF45.1 and Pfs48/45 using the higher resolution structure (Kundu et al., 2018). Binding of 85RF45.1 to Pfs48/45 involved all six CDRs and led to a total buried surface area of 1039 Å^2^ on Pfs48/45 with the heavy chain contributing with 650 Å^2^ and the light chain with 389 Å^2^ (Kundu et al., 2018). 85RF45.1 binds at an edge of the β-sandwich of Pfs48/45 D3, by engaging many residues of the β-strand connecting loops and forming several hydrogen bonds and salt bridges (**Figure 3C**). Sequence analysis showed that three low frequency polymorphisms (I/V349, Q/L355, K/E414) are located within the epitope but 85RF45.1 can recognize single-point mutant proteins representing these polymorphisms with nanomolar affinity (Kundu et al., 2018).

TB31F, a humanized version of the potent transmission-blocking antibody 85RF45.1 has been generated for potential usage as a biologic for malaria interventions (Kundu et al., 2018). The crystal structure of TB31F bound to Pfs48/45 revealed a conserved mode of antigen recognition compared to the parental antibody (Kundu et al., 2018). TB31F retained a similar low nanomolar affinity for antigen binding (~3 nM affinity) and potent transmission-reducing activity in SMFA while showing an improved pH tolerance and thermostability with a ~10°C higher melting temperature.

## Discussion

### Structural Characterization of the 6-Cysteine Proteins

Structural characterization is important for understanding the interactions of the 6-cysteine proteins and how they can be inhibited. Most 6-cysteine protein structures have yet to be solved, including structures of the complete ectodomain of the major vaccine candidates Pfs230, Pfs48/45 and Pfs47. Confirmation of their structures would enable functional epitopes to be further defined, with implications for rational vaccine design. Below we discuss some key advances in structural techniques that could help to structurally characterize the 6-cysteine proteins.

#### AlphaFold for Structure Prediction of 6-Cysteine Proteins

AlphaFold enables prediction of protein structures with atomic accuracy, even in cases where no similar structures have been solved before (Senior et al., 2020; Jumper et al., 2021). It has already been employed to predict structures of *Plasmodium* proteins that are difficult to express (Kaur et al., 2022) and to study the interactions of *Plasmodium* proteins with inhibitors (Chagot et al., 2022). AlphaFold appears capable of predicting *Plasmodium* protein structures with high accuracy (Chagot et al., 2022).

For the 6-cysteine proteins of *P. falciparum*, AlphaFold could be utilized for construct and novel antigen design. For this purpose, AlphaFold predictions could assist in defining domain boundaries to obtain well-folded, soluble protein. In addition, it could simplify the process of structural determination. Due to the low amino acid sequence identity between the 14 different family members (17-36%) and the few available crystal structures, phasing *via* molecular replacement has been challenging for 6-cysteine proteins. Experimental phasing was used to determine the structure of Pf41 (Parker et al., 2015), or in the case of 6-cysteine-antibody complexes initial phases were obtained by molecular replacement using antibody fragments as search models only (Kundu et al., 2018; Lennartz et al., 2018; Singh et al., 2020; Coelho et al., 2021). The 6-cysteine part of these structures were built from scratch by iterative cycles of model building and refinement. Instead, AlphaFold predictions could be valuable search models for molecular replacement to solve the phase problem of structurally uncharacterized 6-cysteine proteins.

**Figure 4:**
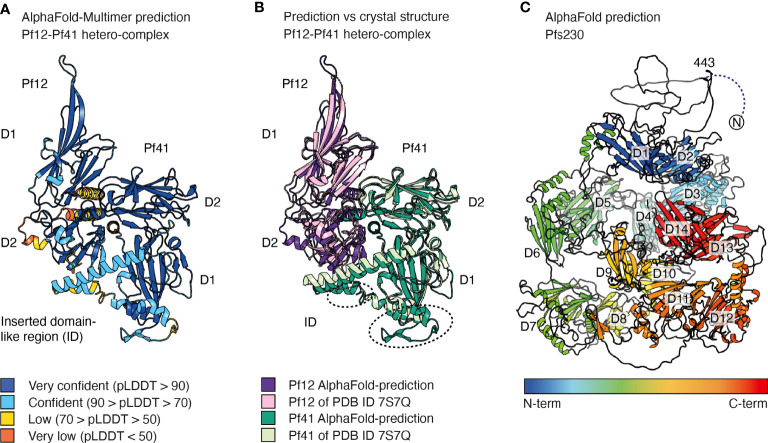
AlphaFold predictions of selected 6-cysteine proteins. **(A)** AlphaFold-Multimer prediction of the hetero-dimeric complex of Pf12 and Pf41. Amino acids are colored based on their per-residue confidence score (pLDDT), which values can be between 0 and 100. Low values indicate low confidence and high numbers indicate very confident predictions. Amino acids are either colored orange (pDLLT <50), yellow (70> pDLLT >50), light blue (50< pDLLT >70), or dark blue (pDLLT >90). Regions with pLDDT <50 may be unstructured in isolation. **(B)** Alignment of the AlphaFold prediction (dark purple and dark green) of the Pf12-Pf41 hetero-dimeric complex with the Pf12-Pf41 crystal structure (light purple and light green, PDB ID 7S7Q). Regions of the Pf41 ID which are not defined in the crystal structure are indicated by dotted circles. **(C)** AlphaFold prediction of Pfs230 colored from blue to red from N- to C-terminus. The N-terminal residues 1-575 have low pDLLT values of <50. Residues 1-442 are not shown for clarity of the image. The 14 6-cysteine domains are indicated from D1-D14. AlphaFold models were generated at WEHI (Australia) using the AlphaFold algorithm.

AlphaFold-Multimer is an AlphaFold model trained on multimeric inputs for the prediction of oligomers and multi-chain protein complexes of known stoichiometry. It could be utilized for *P. falciparum* to provide insights into ligand recognition by 6-cysteine proteins (Evans et al., 2022). This is exemplified by the high accuracy that AlphaFold-Multimer predicts the Pf12-Pf41 hetero-dimer even when excluding the PDB entry of the hetero-complex in the template search of the pre-processing step (**Figure 4A**). The AlphaFold model and the crystal structure of the Pf12-Pf41 hetero-complex overlay with a low r.m.s.d. value of 0.902 Å across 1778 atoms (**Figure 4B**). The core structures of both proteins are predicted with high confidence, as well as most parts of the Pf41 ID. The fold of the Pf41 ID has not been fully characterized by X-ray crystallography, likely due to its flexible nature. In the crystal structure of Pf41 alone the Pf41 ID was likely proteolyzed and therefore mostly absent (PDB 4YS4) (Parker, Peng, and Boulanger 2015). In the crystal structure of the hetero-complex parts of this region were not visible in the electron density and are not connected to the core structure of the 6-cysteine domain (PDB 7S7Q) (Dietrich et al., 2022). Low and very low confidence regions of the AlphaFold model comprise mostly the N-terminal signal sequence of the two proteins and the C-terminal region of Pf12. The arrangement of Pf12 and Pf41 in relation to each other resembles the crystal structure of the complex (Dietrich et al., 2022) and important interactions as shown by mutagenesis studies are predicted within 4 Å. Hence, AlphaFold predicted the overall hetero-complex with high accuracy compared with an experimentally determined structure, and may have shed insights into the fold of a previously uncharacterized region of Pf41. The prediction of hetero-complexes of other 6-cysteine proteins such as P36-P52 or P48/45-P230, or the prediction of 6-cysteine proteins in complex with specific antibodies or nanobodies might be informative but validation with experimental methods remains important.

To date only single and tandem 6-cysteine domains have been structurally characterized by NMR and X-ray crystallography. AlphaFold predictions of larger members of the 6-cysteine protein family will be useful to better understand the arrangement and three-dimensional organization of multiple 6-cysteine domains. For 6-cysteine proteins that contain three 6-cysteine domains, namely P47, P48/45, B9, PSOP12 and P92 (**Figure 1B**), AlphaFold predictions have high and very high per residue confidence scores (pLDDT, predicted local distance difference test) for the core structure of the 6-cysteine domains as well as for most β-strand connecting loops (https://alphafold.ebi.ac.uk/). The models show that all three 6-cysteine domains are highly engaged with each other with many inter-domain contacts between them. The predicted aligned error plot of the AlphaFold models allows assessment of the inter-domain accuracy. Except for Pf92, the expected position error has low values across the whole predicted inter-domain regions of the 6-cysteine domains indicating a high inter-domain accuracy for the 6-cysteine domain arrangement in Pf47, Pfs48/45, PfB9 and PfPSOP12. The proteins Pf92, PfPSOP12 and PfB9 contain in addition to their 6-cysteine domains a predicted N-terminal β-propellor domain which structures are predicted with confidence and have mostly high and very high pLDDT values. The relative position of the β-propellor domains towards the 6-cysteine domains are predicted with confidence for the Pf92 and the PfPSOP12 models. The β-propellor domains were first suggested by structure prediction using HHpred which is now further supported by the AlphaFold predictions.

For Pfs230, the largest member of the family, an AlphaFold prediction shows 14 6-cysteine domains and indicates a mostly unstructured N-terminal pro-domain, where residues 1-575 have very low per-residue confidence scores (**Figure 4C**). Several pro-domain residues form contacts with the first 6-cysteine domain similar to the crystal structures of Pfs230 D1 in complex with antibody fragments (Singh et al., 2020; Coelho et al., 2021). The core structure of the 14 6-cysteine domains and inter-domain regions of A-type and B-type tandem pairs are predicted with high and very high confidence scores. In contrast, several extended loops of the 6-cysteine domains and linker regions between following tandem pairs have low or very low pLDDT values. Some inter-domain contacts are suggested between domains that are not directly adjacent. However, due to the low pLDDT values which could indicate flexibility between tandem domains the model may not allow an accurate description of the inter-domain organization of Pfs230. The predicted model describes one static representation of a possible arrangement of the 14 domains. It remains possible that the packing of the multiple domains is different or requires other interaction partners for its native organization. Similarly, it is possible that the tandem pairs could be mobile relative to each other with no fixed packing, at least in absence of other interaction partners. While the inter-domain arrangement of the Pfs230 model may differ from the native structure, the model provides details about individual tandem pairs of 6-cysteine domains with high confidence and could be valuable for antigen design and as fit and search models for cryo-electron microscopy (cryo-EM) and X-ray crystallography, respectively.

Taken together, structure prediction using AlphaFold will benefit research of 6-cysteine proteins by assisting in construct design, contributing to a better understanding of their fold and interactions with binding partners although validation of the models and their interactions will still require experimental validation.

#### Cryo-EM for Structural Characterization of 6-Cysteine Proteins *In Situ*

Cryo-EM is increasingly used for structural characterization and has been used to solve the structures of key *Plasmodium* proteins at resolutions higher than 4 Å (Kim et al., 2019; Dijkman et al., 2021; Schureck et al., 2021; Lyu et al., 2021). Recent advances have enabled resolutions of under 1.5 Å, allowing direct visualization of atom positions (Nakane et al., 2020; Yip et al., 2020).

Cryo-EM requires less sample than X-ray crystallography and could therefore be particularly valuable for the structural characterization of 6-cysteine proteins that are difficult to express recombinantly. Techniques such as microcrystal electron diffraction (MicroED) allow for structural determination of proteins from as little as a single nanocrystal of protein (Yonekura et al., 2015; Assaiya et al., 2021).

Experimental confirmation of the structure of Pfs230 is important for vaccine design; however, its size complicates expression and crystallization of the full-length protein. The amount of sample required for cryo-EM is sufficiently low to enable structure determination from endogenous *Plasmodium* proteins (Ho et al., 2018; Ho et al., 2021; Anton et al., 2022), which will be useful for proteins such as Pfs230 that cannot be easily expressed recombinantly. Further, with advances in cryo-electron tomography (cryoET) workflows and image processing techniques it is possible to solve the structure of proteins *in situ* at a resolution of 3.5 Å (Wagner et al., 2020; Tegunov et al., 2021). These techniques could be employed to determine the structure of Pfs230 *in situ*, allowing the 14 domains of this protein to be studied in their native conformation.

### Structural Characterization of Inhibitory Antibodies

**Supplementary Table 2** highlights the paucity of reported inhibitory antibodies for several proteins in the 6-cysteine family. Despite the essentiality of at least nine out of 14 P*. falciparum* 6-cysteine proteins, inhibitory mAbs have only been identified for three proteins, namely Pfs230, Pfs48/45 and Pfs47. Generation and characterization of inhibitory antibodies for the remaining proteins could produce valuable research tools and identify additional vaccine candidates. Moreover, there is a lack of structural information for transmission-blocking antibodies in complex with proteins. Structural data for inhibitory mAbs in complex with Pfs48/45 6C and Pfs230 D1 indicate that the mAbs bind to conserved regions of the proteins (Kundu et al., 2018; Lennartz et al., 2018; Singh et al., 2020; Coelho et al., 2021). Structural characterization of additional mAbs interacting with 6-cysteine proteins would aid discovery of conserved binding epitopes and non-competing antibodies that have the potential to act synergistically.

#### Synergistic Antibodies for Investigating and Targeting 6-Cysteine Proteins

Much of the work on 6-cysteine proteins to date has focused on characterizing individual mAbs and there has been little investigation into the potential synergistic effects of antibody combinations. Antibody synergy occurs when the activity of an antibody is enhanced by the presence of other antibodies and can be achieved by combining synergistic mAbs in a cocktail or by including multiple epitopes or antigens in an immunogen to elicit synergistic polyclonal antibodies.

Combining mAbs against different antigens has the potential to increase transmission-blocking activity, as does combining mAbs against different epitopes of the same antigen, which can increase activity through heterotypic interactions (Ragotte et al., 2022). Combining mAbs against different epitopes of Pfs230 D1 or Pfs230 D1 and Pfs48/45 significantly increased transmission-blocking activity (Singh et al., 2020), warranting further investigation of different mAb combinations. Combinations of antibodies with and without transmission-blocking activity may also act synergistically, as was observed for antibodies against PfRh5, where a non-neutralizing antibody was found to enhance the activity of neutralizing antibodies by increasing the time available for them to bind (Alanine et al., 2019). This warrants further characterization of mAbs against 6-cysteine proteins without transmission-blocking or neutralizing activity. The design of bispecific antibodies combining synergistic antibodies targeting multiple epitopes or antigens may enhance activity (Alanine et al., 2019). Synergistic mAbs have therapeutic potential; an antimalarial mAb administered to patients in a Phase I trial demonstrated prophylactic effects (Gaudinski et al., 2021) and treatment with a cocktail of synergistic mAbs could enhance protection.

There are also implications for vaccine design. Targeting multiple epitopes or antigens can induce a synergistic polyclonal response, increasing immune response and reducing the likelihood of escape mutations. A number of 6-cysteine multi-antigen vaccine candidates are under investigation, including Pfs48/45-GLURP (Theisen et al., 2014; Roeffen et al., 2015; Singh et al., 2015; Singh et al., 2017a; Singh et al., 2017b; Singh et al., 2021a), Pfs48/45-GLURP-MSP3 (Baldwin et al., 2016; Mistarz et al., 2017), Pfs230-Pfs48/45 (Singh et al., 2019; Singh et al., 2020; Singh et al., 2021b) and Pfs230-Pfs25 (Menon et al., 2017; Healy et al., 2021). A recent study found a higher prevalence of naturally infected individuals with antibodies against a Pfs48/45-GLURP-MSP3-based vaccine than against the individual proteins, suggesting the combination of antigens has an additive effect on immune response (Baptista et al., 2022). Moreover, animal immunization with the Pfs230-Pfs48/45 chimera elicited transmission-blocking antibody responses three-fold higher than the single antigens alone (Singh et al., 2019), suggesting further investigation of 6-cysteine antigen combinations is warranted.

The epitopes displayed by immunogens should be carefully considered; displaying multiple antigens can elicit antibodies that work synergistically but eliciting a wide polyclonal response can elicit antagonistic antibodies that reduce transmission-blocking activity (Alanine et al., 2019; Ragotte et al., 2022). In addition, synergistic effects appear to be dependent on antigen combination; vaccination with both Pfs230 and Pfs25 was found to elicit antibody responses no higher than those elicited by each antigen individually (Menon et al., 2017; Healy et al., 2021) and combinations of anti-Pfs230 and -Pfs25 mAbs did not show increased transmission-blocking activity (Singh et al., 2020). Identifying antibodies that work synergistically and carefully targeting key epitopes is important for effective malaria control strategies.

## Conclusions

The 6-cysteine proteins are a family of highly conserved, surface exposed proteins expressed throughout the *Plasmodium* life cycle. Structural and functional insights have been derived from mAbs and crystal structures generated using recombinantly expressed 6-cysteine proteins. The structural and functional characterization of Pfs230, Pfs48/45 and Pfs47 as key candidates for transmission-blocking vaccines is a key priority. However, there is still a paucity of structural information for these proteins and their inhibitory antibodies and further characterization, exploiting advances in techniques such as AlphaFold and cryo-EM, would further elucidate the interactions of these proteins and specify functional epitopes for effective targeting of the 6-cysteine proteins.

## Author Contributions

FL, MG, W-HT, and MD wrote the manuscript. All authors contributed to the article and approved the submitted version.

## Funding

W-HT is a Howard Hughes Medical Institute-Wellcome Trust International Research Scholar (208693/Z/17/Z) and supported by National Health and Medical Research Council of Australia (GNT2001385, GNT1154937).

## Conflict of Interest

The authors declare that the research was conducted in the absence of any commercial or financial relationships that could be construed as a potential conflict of interest.

## Publisher’s Note

All claims expressed in this article are solely those of the authors and do not necessarily represent those of their affiliated organizations, or those of the publisher, the editors and the reviewers. Any product that may be evaluated in this article, or claim that may be made by its manufacturer, is not guaranteed or endorsed by the publisher.
