# Supplementary material for: Plasmodium 6-Cysteine Proteins: Functional Diversity, Transmission-Blocking Antibodies and Structural Scaffolds
Source: Front Cell Infect Microbiol. 2022 Jul 8;12:945924. doi: 10.3389/fcimb.2022.945924 (PMC9309271; doi:10.3389/fcimb.2022.945924)
Supplement: Supplementary file 1 [file Table_1.docx]

Supplementary Table 1. Recombinant expression of 6-cysteine proteins.

| **Protein** | **Expression system** | **Species** | **PlasmoDB ID** | **Region** | **Tags and conjugates** | **Downstream applications** | **Reference** | |
| --- | --- | --- | --- | --- | --- | --- | --- | --- |
| P36 | Mammalian | *P. yoelii* | PY17X_1003500 | aa 74-356 (Full ectodomain) | 8xHis | Protein-protein interaction studies | Kaushansky et al. 2015 | |
| P52 | Mammalian | *P. yoelii* | PY17X_1003600 | aa 25-457 (Full ectodomain) | 8xHis | Protein-protein interaction studies | Kaushansky et al. 2015 | |
| P12 | Baculovirus / insect cells | *P. falciparum* | PF3D7_0612700 | aa 28-304 (D1D2) | 8xHis | Protein-protein interaction studies | Dietrich et al. 2021 | |
|  |  |  |  | aa 28-304 (D1D2) | 8xHis | Animal immunization, Immunoreactivity studies, Protein-protein interaction studies, Structural characterization | Dietrich et al. 2022 | |
|  |  |  |  | aa 26-321, NXA (Full ectodomain) | 6xHis + MBP | Protein-protein interaction studies | Parker et al. 2015 | |
|  |  |  |  | aa 28-304 (Pf12short) | 6xHis | Animal immunization, Biochemical characterization, Immunoreactivity studies, Protein-protein interaction studies, Structural characterization | Tonkin et al. 2013 | |
|  |  |  |  | aa 26-321 (Full ectodomain, Pf12long) | 6xHis |  |  |  |
|  |  |  |  | aa 26-321, NXA (Full ectodomain, Pf12long(NXA)) | 6xHis + MBP |  |  |  |
|  | Cell free | *P. vivax* | PVX_113775 | aa 27-336 (Full ectodomain) | GST | Protein-protein interaction studies | Arevalo-Pinzon et al. 2018 | |
|  |  |  |  |  | Halo |  |  |  |
|  |  |  |  | Full ectodomain (aa region unknown) | 6xHis | Immunoreactivity studies | Chen et al. 2010 | |
|  |  |  |  | aa 25-338 (Full ectodomain) | 6xHis | Animal immunization, Immunoreactivity studies | Li et al. 2012 | |
|  | *E. coli* | *P. falciparum* | PF3D7_0612700 | aa 148-274 (D2) | 6xHis | Structural characterization | Arredondo et al. 2012 | |
|  |  |  |  | aa 25-321 (Full ectodomain) | 6xHis or GST | Immunoreactivity studies | Richards et al. 2013 | |
|  |  |  |  | aa 26-321 (Full ectodomain) | 6xHis + Strep II | Animal immunization, Immunoreactivity studies | Taechalertpaisarn et al. 2012 | |
|  |  | *P. vivax* | PVX_113775 | aa 24-340 (Full ectodomain) | 6xHis | Animal immunization, Immunoreactivity studies | Moreno-Perez et al. 2013 | |
|  | *L. lactis* | *P. falciparum* | PF3D7_0612700 | aa 155-323 | 6xHis | Immunoreactivity studies | Kana et al. 2018 | |
|  |  |  |  | aa 155-323 | 6xHis | Biochemical characterization, Immunoreactivity studies | Singh et al. 2018 | |
|  |  |  |  |  | 6xHis + GLURP R0 |  |  |  |
|  | Mammalian | *P. falciparum* | PF3D7_0612700 | aa 26-323, NXA (Full ectodomain) | Cd4d3+4 + Biotinylation sequence | Immunoreactivity studies, Protein-protein interaction studies | Crosnier et al. 2013 | |
|  |  |  |  | aa 26-321, NXA (Full ectodomain) | 6xHis + Cd4d3+4 +  Biotinylation sequence | Protein-protein interaction studies | Taechalertpaisarn et al. 2012 | |
|  |  | *P. vivax* | PVX_113775 | aa 24-339, NXA (Full ectodomain) | Cd4d3+4 + Biotinylation sequence | Immunoreactivity studies, Protein-protein interaction studies | Hostetler et al. 2015 | |
|  |  | *P. falciparum* | PF3D7_0612700 | aa 26-323, NXA (Full ectodomain) | Cd4d3+4 +/- Biotinylation sequence +/- 6xHis | Immunoreactivity studies | Muller-Sienerth et al. 2020 | |
|  |  | *P. vivax* | PVX_113775 | aa 24-339, NXA (Full ectodomain) |  |  |  |  |
|  |  | *P. knowlesi* | PKNH_1137300 | aa 24-323, NXA (Full ectodomain) |  |  |  |  |
| P41 | Baculovirus / insect cells | *P. falciparum* | PF3D7_0404900 | aa 21-368 (D1D2) | 8xHis | Protein-protein interaction studies | Dietrich et al. 2021 | |
|  |  |  |  | aa 21-368 (D1D2) | 8xHis | Animal immunization, Immunoreactivity studies, Protein-protein interaction studies, Structural characterization | Dietrich et al. 2022 | |
|  |  |  |  | aa 21-378, NXA (Full ectodomain) | 6xHis + MBP | Protein-protein interaction studies, Structural characterization | Parker et al. 2015 | |
|  |  |  |  | aa 21-116 + GSGGSG + aa 226-378, NXA (Pf41ΔID) |  |  |  |  |
|  |  |  |  | aa 21-378, NXA (Full ectodomain, Pf41(NXA)) | 6xHis + MBP | Biochemical characterization, Protein-protein interaction studies, Structural characterization | Tonkin et al. 2013 | |
|  | Cell free | *P. vivax* | PVX_000995 | aa 19-383 (Full ectodomain) | GST | Protein-protein interaction studies | Arevalo-Pinzon et al. 2018 | |
|  |  |  |  | Full ectodomain (aa region unknown) | 6xHis | Immunoreactivity studies | Chen et al. 2010 | |
|  |  |  |  | aa 22-384 (Full ectodomain) | 6xHis | Animal immunization, Immunoreactivity studies | Cheng et al. 2013 | |
|  | *E. coli* | *P. falciparum* | PF3D7_0404900 | aa 21-378 (Full ectodomain) | 6xHis or GST | Immunoreactivity studies | Richards et al. 2013 | |
|  |  |  |  | aa 21-378 (Full ectodomain) | 6xHis + Strep II | Animal immunization, Immunoreactivity studies | Taechalertpaisarn et al. 2012 | |
|  | *L. lactis* | *P. falciparum* | PF3D7_0404900 | aa 231-378 | 6xHis + GLURP R0 | Immunoreactivity studies | Kana et al. 2018 | |
|  |  |  |  | aa 231-378 | 6xHis | Biochemical characterization, Immunoreactivity studies | Singh et al. 2018 | |
|  |  |  |  |  | 6xHis + GLURP R0 |  |  |  |
|  | Mammalian | *P. falciparum* | PF3D7_0404900 | aa 21-378, NXA (Full ectodomain) | Cd4d3+4 + Biotinylation sequence | Immunoreactivity studies, Protein-protein interaction studies | Crosnier et al. 2013 | |
|  |  |  |  | aa 21-378, NXA (Full ectodomain) | 6xHis + Cd4d3+4 + Biotinylation sequence | Protein-protein interaction studies | Taechalertpaisarn et al. 2012 | |
|  |  | *P. vivax* | PVX_000995 | aa 22-384, NXA (Full ectodomain) | Cd4d3+4 + Biotinylation sequence | Immunoreactivity studies, Protein-protein interaction studies | Hostetler et al. 2015 | |
|  |  | *P. falciparum* | PF3D7_0404900 | aa 21-378, NXA (Full ectodomain) | Cd4d3+4 +/- Biotinylation sequence +/- 6xHis | Immunoreactivity studies | Muller-Sienerth et al. 2020 | |
|  |  | *P. vivax* | PVX_000995 | aa 22-384, NXA (Full ectodomain) |  |  |  |  |
|  |  | *P. knowlesi* | PKNH_0303000 | aa 22-393, NXA (Full ectodomain) |  |  |  |  |
|  |  | *P. malariae* | PmUG01_03015000 | aa 23-374, NXA (Full ectodomain) |  |  |  |  |
|  |  | *P. ovale curtisi* | PocGH01_03012400 | aa 16-375, NXA (Full ectodomain) |  |  |  |  |
| P12p | Baculovirus / insect cells | *P. falciparum* | PF3D7_0612800 | aa 24-341 (D1D2) | 8xHis | Animal immunization, Immunoreactivity studies, Protein-protein interaction studies, Structural characterization | Dietrich et al. 2021 | |
|  |  |  |  | aa 168-341 (D2) |  |  |  |  |
|  | *E. coli* | *P. falciparum* | PF3D7_0612800 | aa 21-352 (Full ectodomain) | 6xHis or GST | Immunoreactivity studies | Richards et al. 2013 | |
|  | Mammalian | *P. falciparum* | PF3D7_0612800 | aa 21-349, NXA (Full ectodomain) | Cd4d3+4 + Biotinylation sequence | Immunoreactivity studies, Protein-protein interaction studies | Crosnier et al. 2013 | |
|  |  | *P. vivax* | PVX_113780 | aa 24-418, NXA (Full ectodomain) | Cd4d3+4 + Biotinylation sequence | Immunoreactivity studies, Protein-protein interaction studies | Hostetler et al. 2015 | |
|  |  | *P. malariae* | PmUG01_11050400 | aa 19-333, NXA (Full ectodomain) | Cd4d3+4 +/- Biotinylation sequence +/- 6xHis | Immunoreactivity studies | Muller-Sienerth et al. 2020 | |
|  |  | *P. ovale curtisi* | PocGH01_11044100 | aa 23-301, NXA (Full ectodomain) |  |  |  |  |
| P92 | Cell free | *P. falciparum* | PF3D7_1364100 | aa 28-772 (Full ectodomain) | 6xHis or GST | Immunoreactivity studies | Richards et al. 2013 | |
|  | Mammalian | *P. falciparum* | PF3D7_1364100 | aa 26-770, NXA (Full ectodomain) | Cd4d3+4 + Biotinylation sequence | Immunoreactivity studies, Protein-protein interaction studies | Crosnier et al. 2013 | |
|  |  | *P. vivax* | PVX_115165 | aa 23-855, NXA (Full ectodomain) | Cd4d3+4 + Biotinylation sequence | Immunoreactivity studies, Protein-protein interaction studies | Hostetler et al. 2015 | |
|  |  | *P. falciparum* | PF3D7_1364100 | aa 26-770, NXA (Full ectodomain) | Cd4d3+4 +/- Biotinylation sequence +/- 6xHis | Immunoreactivity studies | Muller-Sienerth et al. 2020 | |
|  |  | *P. knowlesi* | PKNH_1107200 | aa 24-857, NXA (Full ectodomain) |  |  |  |  |
| P38 | *E. coli* | *P. falciparum* | PF3D7_0508000 | aa 22-327 (Full ectodomain) | 6xHis or GST | Immunoreactivity studies | Richards et al. 2013 | |
|  | *L. lactis* | *P. falciparum* | PF3D7_0508000 | aa 139-327 | 6xHis | Immunoreactivity studies | Kana et al. 2018 | |
|  |  |  |  | aa 139-327 | 6xHis | Biochemical characterization, Immunoreactivity studies | Singh et al. 2018 | |
|  |  |  |  |  | 6xHis + GLURP R0 |  |  |  |
|  | Mammalian | *P. falciparum* | PF3D7_0508000 | aa 22-328, NXA (Full ectodomain) | Cd4d3+4 + Biotinylation sequence | Immunoreactivity studies, Protein-protein interaction studies | Crosnier et al. 2013 | |
|  |  | *P. vivax* | PVX_097960 | aa 29-334, NXA (Full ectodomain) | Cd4d3+4 + Biotinylation sequence | Immunoreactivity studies, Protein-protein interaction studies | Hostetler et al. 2015 | |
|  |  | *P. falciparum* | PF3D7_0508000 | aa 22-328, NXA (Full ectodomain) | Cd4d3+4 +/- Biotinylation sequence +/- 6xHis | Immunoreactivity studies | Muller-Sienerth et al. 2020 | |
|  |  | *P. vivax* | PVX_097960 | aa 29-334, NXA (Full ectodomain) |  |  |  |  |
|  |  | *P. knowlesi* | PKNH_1025600 | aa 3-367, NXA (Full ectodomain) |  |  |  |  |
|  |  | *P. malariae* | PmUG01_06016900 | aa 1-310, NXA (Full ectodomain) |  |  |  |  |
|  |  | *P. ovale curtisi* | PocGH01_10033500 | aa 25-332, NXA (Full ectodomain) |  |  |  |  |
|  | Plant | *P. falciparum* | PF3D7_0508000 | aa 1-331 (Full ectodomain with signal peptide) | 6xHis | Animal immunization, Immunoreactivity studies | Feller et al. 2013 | |
|  |  |  |  |  | 6xHis + RFP |  |  |  |
| P48/45 | Baculovirus / insect cells | *P. falciparum* | PF3D7_1346700 | aa 1-448 (Full-length protein) | None | Immunoreactivity studies | Kocken et al. 1993 | |
|  |  |  |  | aa 291-427 (6C) | 6xHis | Animal immunization, Biochemical characterization, Immunoreactivity studies | Lee et al. 2020 | |
|  |  |  |  | aa 291-427, N299Q N303D (6C-Mut) |  |  |  |  |
|  |  |  |  | aa 28-427 (Full ectodomain) |  |  |  |  |
|  | Drosophila (S2) | *P. falciparum* | PF3D7_1346700 | aa 291-428 (6C) | C-tag | Animal immunization, Immunoreactivity studies, Structural characterization | Lennartz et al. 2018 | |
|  |  |  |  | aa 159-428 (10C) |  |  |  |  |
|  |  |  |  | aa 27-427 (Full ectodomain) |  |  |  |  |
|  | Cell free | *P. falciparum* | PF3D7_1346700 | aa 28-426 (Full ectodomain) | 6xHis | n/a | Muthui et al. 2021 | |
|  | *Chlamydomonas reinhardtii* | *P. falciparum* | PF3D7_1346700 | aa 178-448 | FLAG | Biochemical characterization, Immunoreactivity studies | Jones et al. 2013 | |
|  | *E. coli* | *P. falciparum* | PF3D7_1346700 | aa 159-426 (10C) | HA + 10xHis | Animal immunization | Agrawal et al. 2019 | |
|  |  |  |  |  | HA + 10xHis + Grl1p |  |  |  |
|  |  |  |  | aa 28-127 (F1) | 6xHis | Animal immunization, Immunoreactivity studies | Cao et al. 2016 | |
|  |  |  |  | aa 108-207 (F2) |  |  |  |  |
|  |  |  |  | aa 188-287 (F3) |  |  |  |  |
|  |  |  |  | aa 268-367 (F4) |  |  |  |  |
|  |  |  |  | aa 348-427 (F5) |  |  |  |  |
|  |  |  |  | aa 28-427 (Full ectodomain) |  |  |  |  |
|  |  |  |  | aa 28-427 (Full ectodomain) | 6xHis | Animal immunization, Immunoreactivity studies | Chowdhury et al. 2009 | |
|  |  |  |  | aa 118-218 | GST | Animal immunization | Kocken et al. 1993 | |
|  |  |  |  | aa 56-401 | GST | Animal immunization, Immunoreactivity studies | Milek, Roeffen et al. 1998 | |
|  |  |  |  | aa 295-427 (6C) | 6xHis | Animal immunization, Biochemical characterization, Immunoreactivity studies | Outchkourov et al. 2007 | |
|  |  |  |  |  | GST |  |  |  |
|  |  |  |  | aa 236-427 (8C) | GST |  |  |  |
|  |  |  |  | aa 173-427 (10C) | 6xHis |  |  |  |
|  |  |  |  |  | GST |  |  |  |
|  |  |  |  | aa 108-427 (12C) | 6xHis |  |  |  |
|  |  |  |  |  | GST |  |  |  |
|  |  |  |  | aa 27-427 (16C) | 6xHis |  |  |  |
|  |  |  |  |  | GST |  |  |  |
|  |  |  |  | aa 27-427, C->S (16 constructs, one of the 16 cysteines mutated in each construct) | GST |  |  |  |
|  |  |  |  | aa 159-428 (10C) | MBP | Animal immunization, Biochemical characterization, Immunoreactivity studies | Outchkourov et al. 2008 | |
|  |  |  |  | aa 26-428 (16C) |  |  |  |  |
|  |  |  |  | aa 28-427 (Full ectodomain) | 6xHis | Animal immunization, Immunoreactivity studies | Pritsch et al. 2016 | |
|  |  |  |  | Domain 3 (D3, aa boundaries unknown) | 6xHis | Animal immunization, Immunoreactivity studies | Singh et al. 2020 | |
|  |  | *P. vivax* | PVX_083235 | Full ectodomain (aa region unknown) | 6xHis + Thioredoxin | Animal immunization, Immunoreactivity studies | Arevalo-Herrera et al. 2015 | |
|  |  |  |  | Full ectodomain (aa region unknown) | 6xHis + Thioredoxin | Immunoreactivity studies | Arevalo-Herrera et al. 2021 | |
|  |  |  |  | Full ectodomain (aa region unknown) | 6xHis + Thioredoxin | Animal immunization, Immunoreactivity studies | Arevalo-Herrera et al. 2022 | |
|  |  |  |  | Full ectodomain (aa region unknown) | 6xHis | Animal immunization, Immunoreactivity studies | Cao et al. 2016 | |
|  | *L. lactis* | *P. falciparum* | PF3D7_1346700 | aa 291-428 (C0) | GLURP.R0 + MSP3 + 6xHis | Immunoreactivity studies | Acquah et al. 2017 | |
|  |  |  |  | aa 287-428 (6C) | 6xHis + MSP3 C-term + GLURP R0 | Animal immunization, Immunoreactivity studies | Baldwin et al. 2016 | |
|  |  |  |  | aa 291-428 (6C) | 6xHis + MSP3 C-term + GLURP R0 | Immunoreactivity studies | Baptista et al. 2022 | |
|  |  |  |  | aa 159-428 (10C) | 6xHis + MSP3 C-term + GLURP R0 | Animal immunization, Biochemical characterization, Immunoreactivity studies | Mistarz et al. 2017 | |
|  |  |  |  | aa 159-428 (10C) | 6xHis + GLURP R0 | Animal immunization, Immunoreactivity studies | Roeffen et al. 2015 | |
|  |  |  |  | aa 287-428 (6Ca) | 6xHis + GLURP R0 | Animal immunization, Immunoreactivity studies | Singh et al. 2015 | |
|  |  |  |  | aa 287-419 (6Cb) |  |  |  |  |
|  |  |  |  | aa 291-428 (6Cc) |  |  |  |  |
|  |  |  |  | aa 291-419 (6Cd) |  |  |  |  |
|  |  |  |  | aa 291-428 (6C) | 6xHis + GLURP R0 | Animal immunization, Biochemical characterization, Immunoreactivity studies | Singh et al. 2019 | |
|  |  |  |  |  | 6xHis + SpyCatcher + P230 Pro (aa 443-590) |  |  |  |
|  |  |  |  |  | 6xHis + SpyCatcher + P230 Pro+1 (aa 443-736) |  |  |  |
|  |  |  |  | aa 291-428 (6C) | GLURP R0 | Animal immunization, Immunoreactivity studies | Singh, Plieskatt, Chourasia, Fabra-Garcia et al. 2021 | |
|  |  |  |  | aa 291-428 (6C) | 6xHis + P230 Pro (aa 443-590) | Animal immunization, Biochemical characterization, Immunoreactivity studies | Singh, Plieskatt, Chourasia, Singh et al. 2021 | |
|  |  |  |  |  | C-tag + P230 Pro (aa 443-590) |  |  |  |
|  |  |  |  | aa 291-428 (6C) | 6xHis + GLURP R0 | Animal immunization, Biochemical characterization, Immunoreactivity studies | Singh, Roeffen et al. 2017 | |
|  |  |  |  |  | 6xHis + GLURP R0 + mSA |  |  |  |
|  |  |  |  |  | 6xHis + GLURP R0 + SpyCatcher |  |  |  |
|  |  |  |  |  | 6xHis + GLURP R0 + SpyTag |  |  |  |
|  |  |  |  |  | GLURP R0 |  |  |  |
|  |  |  |  | aa 291-428 (6C) | 6xHis + GLURP R0 + SpyCatcher | Animal immunization, Immunoreactivity studies | Singh, Thrane et al. 2017 | |
|  |  |  |  |  | 6xHis + SpyCatcher |  |  |  |
|  |  |  |  | aa 159-428 (10C) | 6xHis + GLURP R0 | Animal immunization, Immunoreactivity studies | Theisen et al. 2014 | |
|  | Mammalian | *P. falciparum* | PF3D7_1346700 | aa 291-428 (6C) | 6xHis + GLURP R0 | Protein-protein interaction studies | Kundu et al. 2018 | |
|  |  |  |  |  | 6xHis | Protein-protein interaction studies, Structural characterization |  |  |
|  |  | *P. vivax* | PVX_083235 | Full ectodomain (aa region unknown) | 6xHis + Thioredoxin | Immunoreactivity studies | Arevalo-Herrera et al. 2021 | |
|  |  |  |  | Full ectodomain (aa region unknown) | 6xHis + Thioredoxin | Animal immunization, Immunoreactivity studies | Arevalo-Herrera et al. 2022 | |
|  | Plant | *P. falciparum* | PF3D7_1346700 | aa 28-401 (Full ectodomain) | 6xHis | Biochemical characterization, Immunoreactivity studies | Mamedov et al. 2012 | |
|  |  |  |  | aa 159-401 (10C) | FLAG | Biochemical characterization, Immunoreactivity studies | Mamedov et al. 2017 | |
|  |  |  |  | aa 28-401 (Full ectodomain) |  |  |  |  |
|  |  |  |  | aa 159-428 (10C) | FLAG | Animal immunization, Biochemical characterization, Immunoreactivity studies | Mamedov et al. 2019 | |
|  |  |  |  | aa 28-428 (Full ectodomain) |  |  |  |  |
|  |  |  |  | aa 28-401 | 6xHis | Biochemical characterization | Prokhnevsky et al. 2015 | |
|  | Yeast | *P. falciparum* | PF3D7_1346700 | aa 1-399, N->Q | None | Animal immunization, Immunoreactivity studies | Milek et al. 2000 | |
|  |  |  |  | aa 1-448, N->Q (Full-length protein) |  |  |  |  |
|  |  |  |  | aa 28-399, N->Q | His |  |  |  |
|  |  |  |  | aa 28-349, NXA (D1-D2) | 6xHis | Animal immunization | Singh et al. 2020 | |
| P230 | Baculovirus / insect cells | *P. falciparum* | PF3D7_0209000 | aa 443-731, N585Q (Pfs230C1) | 6xHis | Animal immunization, Immunoreactivity studies | Huang et al. 2020 | |
|  |  |  |  | aa 443-731, N585Q (Pfs230C1) | 6xHis | Animal immunization, Immunoreactivity studies | Lee et al. 2017 | |
|  |  |  |  | aa 443-731, N585Q (Pfs230C1) | 6xHis | Animal immunization, Immunoreactivity studies | Lee, Plieskatt et al. 2019 | |
|  |  |  |  | aa 552-731, N585Q (Pfs230D1+) | 6xHis | Animal immunization, Biochemical characterization, Immunoreactivity studies | Lee, Wu et al. 2019 | |
|  |  |  |  | aa 443-731 (Pfs230C1), N585Q | 6xHis | Animal immunization, Immunoreactivity studies | Miura et al. 2019 | |
|  |  |  |  |  | 6xHis + CRM197 |  |  |  |
|  | Cell free | *P. falciparum* | PF3D7_0209000 | aa 443-1132 (Pfs230C) | 6xHis | Animal immunization, Immunoreactivity studies | Miura et al. 2013 | |
|  |  |  |  | aa 443 – 588 (cPro) | 6xHis | Animal immunization, Immunoreactivity studies | Miura et al. 2022 | |
|  |  |  |  | aa 443-918 (cPro/CM1-2) |  |  |  |  |
|  |  |  |  | aa 543-588 (shPro) |  |  |  |  |
|  |  |  |  | aa 543-731 (shPro/CM1) |  |  |  |  |
|  |  |  |  | aa 918-1274 (CM3-4) |  |  |  |  |
|  |  |  |  | aa 543-588 (shPro) | KLH |  |  |  |
|  |  |  |  | aa 443-1132 (Pfs230C) | 6xHis | Immunoreactivity studies | Muthui et al. 2021 | |
|  |  |  |  | aa 443-1132 (Pfs230C) | GST | Animal immunization, Immunoreactivity studies | Tachibana et al. 2011 | |
|  |  |  |  | aa 443-588 (Pfs230C0) |  |  |  |  |
|  |  |  |  | aa 443-715 (Pfs230C1) |  |  |  |  |
|  |  |  |  | aa 443-915 (Pfs230C2) |  |  |  |  |
|  |  |  |  | aa 443-1274 (TBV01, CM1-4) | 6xHis | Animal immunization, Immunoreactivity studies | Tachibana et al. 2019 | |
|  |  |  |  | aa 1280-2051 (TBV02, CM5-8) |  |  |  |  |
|  |  |  |  | aa 2052-2830 (TBV03, CM9-12) |  |  |  |  |
|  |  |  |  | aa 2448-3135 (TBV04, CM11-14) |  |  |  |  |
|  |  |  |  | aa 443-904 (TBV05, CM1-2) |  |  |  |  |
|  |  |  |  | aa 910-1274 (TBV06, CM3-4) |  |  |  |  |
|  |  |  |  | aa 1280-1560 (TBV07, CM5-6) |  |  |  |  |
|  |  |  |  | aa 1690-2051 (TBV08, CM7-8) |  |  |  |  |
|  |  |  |  | aa 2052-2393 (TBV09, CM9-10) |  |  |  |  |
|  |  |  |  | aa 2448-2830 (TBV10, CM11-12) |  |  |  |  |
|  |  |  |  | aa 2831-3135 (TBV11, CM13-14) |  |  |  |  |
|  |  |  |  | aa 443-730 (TBV12, CM1) |  |  |  |  |
|  |  |  |  | aa 731-904 (TBV13, CM2) |  |  |  |  |
|  |  |  |  | aa 910-1133 (TBV14, CM3) |  |  |  |  |
|  |  |  |  | aa 1134-1274 (TBV15, CM4) |  |  |  |  |
|  |  |  |  | aa 1280-1432 (TBV16, CM5) |  |  |  |  |
|  |  |  |  | aa 1433-1560 (TBV17, CM6) |  |  |  |  |
|  |  |  |  | aa 1690-1907 (TBV18, CM7) |  |  |  |  |
|  |  |  |  | aa 1908-2051 (TBV19, CM8) |  |  |  |  |
|  |  |  |  | aa 2052-2201 (TBV20, CM9) |  |  |  |  |
|  |  |  |  | aa 2202-2393 (TBV21, CM10) |  |  |  |  |
|  |  |  |  | aa 2448-2663 (TBV22, CM11) |  |  |  |  |
|  |  |  |  | aa 2664-2830 (TBV23, CM12) |  |  |  |  |
|  |  |  |  | aa 2831-2979 (TBV24, CM13) |  |  |  |  |
|  |  |  |  | aa 2980-3135 (TBV25, CM14) |  |  |  |  |
|  |  |  |  | aa 543-730 (TBV26, CM1) |  |  |  |  |
|  |  |  |  | aa 443-1132 (TBV27, CM1-3) |  |  |  |  |
|  |  |  |  | aa 910-1560 (TBV29, CM3-6) |  |  |  |  |
|  |  |  |  | aa 1690-2393 (TBV30, CM7-10) |  |  |  |  |
|  |  |  |  | aa 22-588 (TBV31, Prodomain) |  |  |  |  |
|  | *E. coli* | *P. falciparum* | PF3D7_0209000 | aa 443-1132 (region C) | 6xHis + MBP | Animal immunization, Immunoreactivity studies | Bustamante et al. 2000 | |
|  |  |  |  | aa 443-791 (C5’) |  |  |  |  |
|  |  |  |  | aa 453-913 (C1.6) |  |  |  |  |
|  |  |  |  | aa 583-913 (CM1) |  |  |  |  |
|  |  |  |  | aa 914-1268 (CM2) |  |  |  |  |
|  |  |  |  | aa 443-915 (Pfs230D1-2) | 6xHis | Animal immunization, Biochemical characterization, Immunoreactivity studies | MacDonald et al. 2016 | |
|  |  |  |  | Tetrapeptide repeat region (r230-1, aa region unknown) | MBP | Immunoreactivity studies | Riley et al. 1995 | |
|  |  |  |  | CR1-CR2 (r230-2, aa region unknown) |  |  |  |  |
|  |  |  |  | CR2-CR3 (r230-3, aa region unknown) |  |  |  |  |
|  |  |  |  | CR4-CR5 (r230-4, aa region unknown) |  |  |  |  |
|  |  |  |  | CR6-CR7 (r230-5, aa region unknown) |  |  |  |  |
|  |  |  |  | Poly-glutamate region (r230-r2, aa region unknown) |  |  |  |  |
|  |  |  |  | aa 443-592 (Pfs230NS) | 6xHis | Immunoreactivity studies | Singh et al. 2020 | |
|  |  |  |  | Full-length protein (aa region unknown) | MBP | Animal immunization, Immunoreactivity studies | Williamson et al. 1993 | |
|  |  |  |  | aa 304-378 (r230/MBP.A) | MBP | Animal immunization, Immunoreactivity studies | Williamson et al. 1995 | |
|  |  |  |  | aa 375-452 (r230/MBP.B) |  |  |  |  |
|  |  |  |  | aa 443-1132 (r230/MBP.C) |  |  |  |  |
|  |  |  |  | aa 1166-1541 (r230/MBP.D) |  |  |  |  |
|  |  |  |  | aa 1727-2397 (r230/MBP.E) |  |  |  |  |
|  |  |  |  | aa 2398-3135 (r230/MBP.F) |  |  |  |  |
|  | *L. lactis* | *P. falciparum* | PF3D7_0209000 | aa 443-590 (6C) | 6xHis | Immunoreactivity studies | Acquah et al. 2017 | |
|  |  |  |  | aa 443-1132 (Pro+I,II,III) | 6xHis + SpyCatcher | Animal immunization, Biochemical characterization, Immunoreactivity studies | Singh et al. 2019 | |
|  |  |  |  | aa 443-590 (Pro) | 6xHis + SpyCatcher |  |  |  |
|  |  |  |  | aa 443-590 (Pro) | 6xHis + SpyCatcher + P48/45 6C (aa 291-428) |  |  |  |
|  |  |  |  | aa 443-736 (Pro+I) | 6xHis + SpyCatcher |  |  |  |
|  |  |  |  | aa 443-736 (Pro+I) | 6xHis + SpyCatcher + P48/45 6C (aa 291-428) |  |  |  |
|  |  |  |  | aa 443-590 (Pro) | 6xHis + P48/45 6C | Animal immunization, Biochemical characterization, Immunoreactivity studies | Singh, Plieskatt, Chourasia, Singh et al. 2021 | |
|  |  |  |  |  | C-tag + P48/45 6C |  |  |  |
|  | Mammalian | *P. falciparum* | PF3D7_0209000 | aa 542-736, N585Q (Pfs230D1M) | 6xHis | Immunoreactivity studies | Chan et al. 2019 |  |
|  | Plant | *P. falciparum* | PF3D7_0209000 | aa 444-730 (230CMB) | 6xHis | Animal immunization, Biochemical characterization, Immunoreactivity studies | Farrance et al. 2011 | |
|  | Yeast | *P. falciparum* | PF3D7_0209000 | aa 443-1132 (Pfs230c) | dS | Animal immunization, Immunoreactivity studies | Chan et al. 2019 | |
|  |  |  |  | aa 542-736 (Pfs230D1M) |  |  |  |  |
|  |  |  |  | aa 542-736 (Pfs230D1) | EPA | Animal immunization, Immunoreactivity studies | Coelho et al. 2019 | |
|  |  |  |  | aa 542-736 (Pfs230D1) | Biotinylation sequence | Human immunization, Immunoreactivity studies, Structural characterization | Coelho et al. 2021 | |
|  |  |  |  |  | EPA |  |  |  |
|  |  |  |  |  | None |  |  |  |
|  |  |  |  | aa 542-736 (Pfs230D1M) | EPA | Animal immunization, Human immunization, Immunoreactivity studies | Healy et al. 2021 | |
|  |  |  |  | aa 444-736, N585Q (Pfs230D1H) | 6xHis | Animal immunization, Biochemical characterization, Immunoreactivity studies | MacDonald et al. 2016 | |
|  |  |  |  | aa 542-736, N585Q (Pfs230D1M) | None |  |  |  |
|  |  |  |  | aa 542-736, T587A (Pfs230D1A) | 6xHis | Immunoreactivity studies, Structural characterization | Singh et al. 2020 | |
|  |  |  |  | aa 542-736, N585Q (Pfs230D1M) |  |  |  |  |
|  |  |  |  | aa 443-1132 (Pfs230c) | dS | Biochemical characterization | Wetzel et al. 2019 | |
|  |  |  |  | aa 542-736 (Pfs230D1M) |  |  |  |  |
| P47 | Baculovirus / insect cells | *P. falciparum* | PF3D7_1346800 | aa 32-154 + GSGGSG + aa 268-414 (D1 + D3) | 6xHis | Animal immunization, Immunoreactivity studies | Canepa et al. 2018 | |
|  |  |  |  | aa 32-420 (Full ectodomain) | 6xHis + MBP |  |  |  |
|  |  |  |  | aa 32-420 (Full ectodomain) | 6xHis + MBP | Protein-protein interaction studies | Molina-Cruz et al. 2020 | |
|  | *E. coli* | *P. falciparum* | PF3D7_1346800 | aa 155-267, C230A and C260A (mD2) | 6xHis | Animal immunization, Immunoreactivity studies | Canepa et al. 2018 | |
|  |  |  |  | aa 178-267, C230A and C260A (mD2-Del1) |  |  |  |  |
|  |  |  |  | aa 178-229 (mD2-Del2) |  |  |  |  |
|  |  |  |  | aa 155-181 (mD2-Del3) |  |  |  |  |
|  |  |  |  | aa 32-154 (D1) | 6xHis + Thioredoxin |  |  |  |
|  |  |  |  | aa 268-414 (D3) |  |  |  |  |
|  |  |  |  | aa 32-420 (Full ectodomain) |  |  |  |  |
|  |  |  |  | aa 178-235 | 6xHis + SpyTag | Animal immunization, Immunoreactivity studies | Yenkoidiok-Douti et al. 2019 | |
|  |  |  |  | aa 178-229 | 6xHis | Animal immunization, Immunoreactivity studies | Yenkoidiok-Douti et al. 2021 | |
|  |  | *P. berghei* | PBANKA_1359700 | aa 21-412 (Full ectodomain) | 6xHis + Thioredoxin | Animal immunization, Antibody generation | Ukegbu et al. 2017 | |
|  |  |  |  | D1 | 6xHis | Animal immunization, Immunoreactivity studies | Yenkoidiok-Douti et al. 2020 | |
|  |  |  |  | aa 129-237, C202A, C232A (mD2) |  |  |  |  |
|  |  |  |  | aa 152-237, C202A, C232A (Del1) |  |  |  |  |
|  |  |  |  | D3 |  |  |  |  |
|  |  |  |  | aa 152-237, C202A, C232A (Del1) | 6xHis + SpyTag |  |  |  |
|  |  |  |  | aa 22-380 (Pbs47-FL) | 6xHis + Thioredoxin |  |  |  |
|  | Mammalian | *P. falciparum* | PF3D7_1346800 | aa 27-415 (Full ectodomain) | 6xHis | n/a | Muthui et al. 2021 | |
| PSOP12 | Baculovirus / insect cells | *P. berghei* | PBANKA_1113400 | aa 294-721, N337Q, N608Q, N700Q | FLAG + VSV-G | Animal immunization, Immunoreactivity studies | Sala et al. 2015 | |
|  | Mammalian | *P. falciparum* | PF3D7_0513700 | aa 24-735 (Full ectodomain) | 6xHis | Immunoreactivity studies | Muthui et al. 2021 | |

CR, cysteine rich motif; CXS, cysteine residues mutated to serine; dS, duck hepatitis B virus small surface protein; EPA, exoprotein A; GLURP, glutamate-rich protein; GST, glutathione-S- transferase; His, histidine; KLH, keyhole limpet hemocyanin; MBP, maltose-binding protein; mSA, monomeric streptavidin; MSP3, merozoite surface protein 3; NXA, serine or threonine mutated to alanine at NXS/T sites; RFP, red fluorescent protein; VSV-G, vesicular stomatitis virus G.
