# Supplementary material for: Plasmodium 6-Cysteine Proteins: Functional Diversity, Transmission-Blocking Antibodies and Structural Scaffolds
Source: Front Cell Infect Microbiol. 2022 Jul 8;12:945924. doi: 10.3389/fcimb.2022.945924 (PMC9309271; doi:10.3389/fcimb.2022.945924)
Supplement: Supplementary file 2 [file Table_2.docx]

Supplementary Table 2. Monoclonal transmission-blocking antibodies generated against 6-cysteine proteins

| **6-cys Protein** | **PlasmoDB ID** | **Antibody name(s)** | **Isotype** | **Source** | **Transmission Reducing Activity (TRA)** | **Complement dependency** | **Antigen detection methods** | **Epitope domain** | | **Antigen source** | **Antigen description** | **Reference** |
| --- | --- | --- | --- | --- | --- | --- | --- | --- | --- | --- | --- | --- |
| Pfs230 | PD3D7_0209000 | 1A3-B8* | IgG2a | Mouse | 95.8% at 3-8 mg/mL | Dependent** | WB, IP & IFA | | - | Parasite material | *P. falciparum* gametocytes and gametes (mixed isolates) | Rener et al. 1983 |
|  |  | 11C5-B10* | IgG2a | Mouse | 56.3% at 1-3 mg/mL | Dependent | WB, IP & IFA | | - | Parasite material | *P. falciparum* gametocytes and gametes (mixed isolates) | Rener et al. 1983 |
|  |  | 29F432* | IgG | Mouse | 97% at 1-3 mg/mL | Independent | IP | | - | Parasite material | *P. falciparum* macrogametes/zygotes | Vermeulen et al. 1985 |
|  |  | 1B3 | IgG2a | Mouse | 94.0-97.7%, unknown concentration | Dependent | WB, IP & IFA | | - | Parasite material | *P. falciparum* 3D7 macrogametes and zygotes | Williamson et al. 1995 |
|  |  |  |  |  | 95-100% at 100 µg/mL |  |  |  |  |  |  | Quakyi et al. 1987 |
|  |  | 2B4 | IgG2a | Mouse | 95-100% at 100 µg/mL | Dependent | WB, IP & IFA | | - | Parasite material | *P. falciparum* 3D7 macrogametes and zygotes | Quakyi et al. 1987 |
|  |  | 1H2 | IgG2a | Mouse | 100%, unknown concentration | Dependent | WB, IP & IFA | | - | Parasite material | *P. falciparum* 3D7 gametes | Roeffen, Geeraedts et al. 1995 |
|  |  |  |  |  | >99%, unknown concentration |  |  |  |  |  |  | Read et al. 1994 |
|  |  | 3G9 | IgG2a | Mouse | 100%, unknown concentration | Dependent | WB, IP & IFA | | - | Parasite material | *P. falciparum* 3D7 gametes | Roeffen, Geeraedts et al. 1995 |
|  |  |  |  |  | 100%, unknown concentration |  |  |  |  |  |  | Read et al. 1994 |
|  |  | 4C10 | IgG2a | Mouse | 100%, unknown concentration | Dependent | WB, IP & IFA | | - | Parasite material | *P. falciparum* 3D7 gametes | Roeffen, Geeraedts et al. 1995 |
|  |  |  |  |  | 100%, unknown concentration |  |  |  |  |  |  | Read et al. 1994 |
|  |  | 7A6 | IgG2a | Mouse | 100%, unknown concentration | Dependent | WB, IP & IFA | | - | Parasite material | *P. falciparum* 3D7 gametes | Roeffen, Geeraedts et al. 1995 |
|  |  |  |  |  | 100%, unknown concentration |  |  |  |  |  |  | Read et al. 1994 |
|  |  | 8C11 | IgG2a | Mouse | 100%, unknown concentration | Dependent | WB, IP & IFA | | - | Parasite material | *P. falciparum* 3D7 gametes | Roeffen, Geeraedts et al. 1995 |
|  |  |  |  |  | 99-100%, unknown concentration |  |  |  |  |  |  | Read et al. 1994 |
|  |  | 11E3 | IgG2a | Mouse | 100%, unknown concentration | Dependent | WB, IP & IFA | | - | Parasite material | *P. falciparum* 3D7 gametes | Roeffen, Geeraedts et al. 1995 |
|  |  |  |  |  | >99% with 5µg mAb |  |  |  |  |  |  | Roeffen, Beckers et al. 1995 |
|  |  |  |  |  | 100%, unknown concentration |  |  |  |  |  |  | Read et al. 1994 |
|  |  | 12F10 | IgG2a | Mouse | 100%, unknown concentration | Dependent | WB, IP & IFA | | - | Parasite material | *P. falciparum* 3D7 gametes | Roeffen, Geeraedts et al. 1995 |
|  |  |  |  |  | >99% with 5µg mAb |  |  |  |  |  |  | Roeffen, Beckers et al. 1995 |
|  |  |  |  |  | 100%, unknown concentration |  |  |  |  |  |  | Read et al. 1994 |
|  |  | 17E9 | IgG2a | Mouse | 100%, unknown concentration | Dependent | WB, IP & IFA | | - | Parasite material | *P. falciparum* 3D7 gametes | Roeffen, Geeraedts et al. 1995 |
|  |  |  |  |  | 100%, unknown concentration |  |  |  |  |  |  | Read et al. 1994 |
|  |  | 11C12F7 | IgG2a | Mouse | 100%, unknown concentration | Dependent | - | | - | Parasite material | *P. falciparum* 3D7 gametes | Roeffen, Geeraedts et al. 1995 |
|  |  | 12A1A5 | IgG2b | Mouse | 100%, unknown concentration | Dependent | - | | - | Parasite material | *P. falciparum* 3D7 gametes | Roeffen, Geeraedts et al. 1995 |
|  |  | 21C1 | IgG2a | Mouse | 100%, unknown concentration | Dependent | - | | - | Parasite material | *P. falciparum* 3D7 gametes | Roeffen, Geeraedts et al. 1995 |
|  |  | 63F2A2 | IgG2a | Mouse | >99% with 5µg mAb | Dependent | - | | - | Parasite material | Pfs230-enriched aqueous phase extract of NF54 gametocytes | Roeffen, Beckers et al. 1995 |
|  |  | 63F2A2.2a | IgG2a | Mouse | SMFA IC80 of 1.9 µg/mL,  DMFA IC80 15 µg/mL | Dependent | WB, radiolabeled microscopy, imaging-based complement lysis assay & IFA | | - | Parasite material | Pfs230-enriched aqueous phase extract of NF54 gametocytes | de Jong et al. 2021 |
|  |  |  |  |  | >99% at >4 µg/mL, 80.1% at 0.8 µg/mL |  |  |  |  |  |  | Roeffen Geeraedts et al. 1995 |
|  |  | 63F2A2.2b | IgG2b | Mouse | >90% at >20 µg/mL, 70.5% at 4 µg/mL | Dependent | WB & radiolabeled microscopy | | - | Parasite material | Pfs230-enriched aqueous phase extract of gametocytes | Roeffen Geeraedts et al. 1995 |
|  |  | 4F12 | IgG1 | Mouse | 99.6-100% at 1 mg/mL | Independent | WB, IFA | | Domain 1 | Parasite material | *P. falciparum* female gametocytes | MacDonald et al. 2016 |
|  |  |  |  |  | 40.3% at 1.1mg/mL, 42.2-67.1% at 1mg/mL |  |  |  |  |  |  | Singh et al. 2020 |
|  |  | rh4F12 | mFab/hIgG1 Fc | Chimeric human/mouse 4F12 antibody | 92.2% at 1mg/ml, 92.3% at 630 μg/mL, 84.3% at 600 μg/mL | Independent | WB & IFA | | Domain 1 | Parasite material | *P. falciparum* female gametocytes | Singh et al. 2020 |
|  |  | 5H1 | IgG1 | Mouse | 92.9-100% at 1 mg/mL, 24.8% at 630 μg/mL, 65.1% at 250 μg/mL | Dependent | IFA | | Domain 1 | Recombinant protein | Pfs230 aa 542-736, N585Q (Pfs230D1M) | Singh et al. 2020 |
|  |  | LMIV230-01 | IgG1 | Human (memory B cells) | 91.7% at 1 mg/mL, 80.3% at 60 μg/mL | Dependent** | WB, IFA & imaging based-complement lysis assay | | Domain 1 | Recombinant protein | Pfs230 aa 542-736 + EPA (Pfs230D1-EPA) | Coelho et al. 2021 |
|  |  | LMIV230-02 | IgG1 | Human (memory B cells) | 58.7% at 1 mg/mL | Dependent | WB | | Domain 1 | Recombinant protein | Pfs230 aa 542-736 + EPA (Pfs230D1-EPA) | Coelho et al. 2021 |
| Pfs48/45 | PF3D7_1346700 | 1A3-B8* | IgG2a | Mouse | 95.8% at 3-8 mg/mL | Dependent** | WB, IP & IFA | | - | Parasite material | *P. falciparum* gametocytes and gametes (mixed isolates) | Rener et al. 1983 |
|  |  | 11C5-B10* | IgG2a | Mouse | 56.3% at 1-3 mg/mL | Dependent | WB, IP & IFA | | - | Parasite material | *P. falciparum* gametocytes and gametes (mixed isolates) | Rener et al. 1983 |
|  |  | 29F432* | IgG | Mouse | 97% at 1-3 mg/mL | Independent | IP | | - | Parasite material | *P. falciparum* macrogametes/zygotes | Vermeulen et al. 1985 |
|  |  | 32F3 | IgG2b | Mouse | SMFA IC80 of 1.8 μg/mL in SMFA, DMFA IC80 of at 1.7-2.1 μg/mL (isolate dependent) | Independent | WB, IP & IFA | | Epitope I (C-terminal domain) | Parasite material | *P. falciparum* macrogametes/zygotes | de Jong et al. 2021 |
|  |  |  |  |  | 99.8% at 350 μg/mL |  |  |  |  |  |  | Lennartz et al. 2018 |
|  |  |  |  |  | 99.8% at 50 μg/mL, 98.9% at 25 μg/mL, 61.4% at 12.5 μg/mL |  |  |  |  |  |  | Roeffen, Teelen et al. 2001 |
|  |  |  |  |  | 100% at 1-3 mg/mL |  |  |  |  |  |  | Vermeulen et al. 1985 |
|  |  | 32F5 | IgG2b | Mouse | 95% at 1-3 mg/mL | Independent | IP | | Epitope I (C-terminal domain) | Parasite material | *P. falciparum* macrogametes/zygotes | Vermeulen et al. 1985 |
|  |  | 42A6-F3 | IgG1 | Mouse | 84.0%, unknown concentration | Independent | - | | Epitope II (central domain) | Parasite material | Affinity purified *P. falciparum* Pfs48/45 | Targett et al. 1988 |
|  |  | 81D3-D2 | IgM | Mouse | 99.4%, unknown concentration | Independent | - | | Epitope III (central domain) | Parasite material | Affinity purified *P. falciparum* Pfs48/45 | Targett et al. 1988 |
|  |  | 82C4-A9 | IgM | Mouse | 99.9%, unknown concentration | Independent | - | | Epitope III (central domain) | Parasite material | Affinity purified *P. falciparum* Pfs48/45 | Targett et al. 1988 |
|  |  | 84A2-A4 | IgG2a | Mouse | 100%, unknown concentration | Independent | - | | Epitope V (N-terminal domain) | Parasite material | Affinity purified *P. falciparum* Pfs48/45 | Targett et al. 1988 |
|  |  | 82D6-A10 | IgG2a | Mouse | 94.3%, unknown concentration | Independent | - | | Epitope II (central domain) | Parasite material | Affinity purified *P. falciparum* Pfs48/45 | Targett et al. 1988 |
|  |  | 3E12 | IgG1 | Mouse | 70.8-82.6% at 40 μg/mL | Independent | WB & IFA | | Epitope I (C-terminal domain) | Parasite material | *P. falciparum* 7G8 gametes | Singh et al 2020 |
|  |  |  |  |  | >98% at >40 μg/mL for intact mAb and at >24 μg/mL for Fab fragment |  |  |  |  |  |  | Carter et al. 1990 |
|  |  | 85RF45.1 | IgG1 | Rat | SMFA IC80 of 1.8 μg/mL in SMFA, DMFA IC80 of at 1.7-2.1 μg/mL (isolate dependent) | - | WB & IFA | | Epitope I (C-terminal domain) | Parasite material | *P. falciparum* gametocytes | de Jong et al. 2021 |
|  |  |  |  |  | 100% at 14 μg/mL |  |  |  |  |  |  | Lennartz et al. 2018 |
|  |  |  |  |  | 80% at ~1.0-2.0 μg/mL |  |  |  |  |  |  | Kundu et al. 2018 |
|  |  |  |  |  | 100% at 12.5 μg/mL |  |  |  |  |  |  | Roeffen, Teelen et al. 2001 |
|  |  | TB31F | hIgG1 | Humanized 85RF45.1 antibody | 80% at ~0.5-1.0 μg/mL | - | - | | Epitope I (C-terminal domain) | Parasite material | *P. falciparum* gametocytes | Kundu et al. 2018 |
|  |  | 85RF45.5 | IgG2a | Rat | 99.1% at 100 μg/mL, 98.1% at 50 μg/mL, 79.1% at 25 μg/mL | - | WB & IFA | | Epitope V (N-terminal domain) | Parasite material | *P. falciparum* gametocytes | Roeffen, Teelen et al. 2001 |
|  |  | 1F10 | IgG1 | Mouse | 74.5% at 375 μg/mL, 61.0% at 350 μg/mL | - | IFA | | Epitope IIb/III (central domain) | Recombinant protein | Pfs48/45 aa 27-427 (Full ectodomain) | Lennartz et al. 2018 |
|  |  | 3G3 | IgG2a | Mouse | 59.1% at 375 μg/mL | - | IFA | | Epitope IIb/III (central domain) | Recombinant protein | Pfs48/45 aa 27-427 (Full ectodomain) | Lennartz et al. 2018 |
|  |  | 6A10 | IgG1 | Mouse | 74.5% at 375 μg/mL | - | IFA | | Epitope IIb/III (central domain) | Recombinant protein | Pfs48/45 aa 27-427 (Full ectodomain) | Lennartz et al. 2018 |
|  |  | 10D8 | IgG1 | Mouse | 55.9% at 375 μg/mL | - | IFA | | Epitope IIb/III (central domain) | Recombinant protein | Pfs48/45 aa 27-427 (Full ectodomain) | Lennartz et al. 2018 |
| Pfs47 | PF3D7_1346800 | IB2 | - | Mouse | 99% at 200µg/mL | Independent | - | | Domain 2 | Recombinant protein | Pfs47 aa 155-267, C230A and C260A (mD2) | Canepa et al. 2018 |
|  |  | BM2 | - | Mouse | 81% at 200μg/mL | Independent | - | | Domain 2 | Recombinant protein | Pfs47 aa 155-267, C230A and C260A (mD2) | Canepa et al. 2018 |

GPI; glycosylphosphatidylinositol; IFA, immunofluorescence assay; IP, immunoprecipitation; MBP, maltose binding protein; SMFA, standard membrane feeding assay; TRA, transmission reducing activity; WB, western blotting.

% TRA is defined as a reduction in the mean number of oocysts/mosquito when assayed by SMFA in the presence of the antibodies.

* This mAb recognizes more than one protein

** Complement dependent but retained some level of inhibition in its absence.
